# A general and green approach of pH‐sensitive ZIF‐67 for encapsulating active substances

**DOI:** 10.1002/smo2.70040

**Published:** 2026-03-11

**Authors:** Xinyi Sun, Yan Qi, Xinhao Che, Lulu Zhou, Yuxin Li, Lei Zhang, Jing Hu

**Affiliations:** ^1^ School of Perfume and Aroma Technology Shanghai Institute of Technology Shanghai China; ^2^ School of Chemical Engineering Dalian University of Technology Dalian China; ^3^ School of Chemical Engineering East China University of Science and Technology Shanghai China

**Keywords:** delivery, green synthesis, molecular simulations, one‐pot, universality, zeolitic imidazolate framework‐67 (ZIF‐67)

## Abstract

Zeolitic imidazolate framework‐67 (ZIF‐67) has emerged as a promising carrier for active substances (AS) due to its biocompatibility and acid‐triggered degradation behavior. Conventional loading strategies often involve multi‐step post‐synthetic modifications or one‐pot processes employing toxic organic solvents. This study presents a facile one‐pot synthesis method for encapsulating AS into ZIF‐67 in deionized water within 2 h under ambient conditions. This method exhibits broad applicability, enabling effective encapsulation of both hydrophobic and hydrophilic molecules with molecular weights ranging from low to high, with loading efficiencies between 12.17% and 56.11%. While free compounds were released rapidly in a pH‐independent manner, both CA@ZIF‐67 and EGCG@ZIF‐67 proved pH‐responsive sustained release profiles, with their mechanisms best described by the first‐order model and Fickian diffusion, respectively. Moreover, these composites demonstrated synergistic and pH‐dependent antibacterial activity against *Escherichia coli* and *Staphylococcus aureus*, showing significantly enhanced efficacy in acidic conditions. Furthermore, the formation process, morphological evolution, and release behavior of AS@ZIF‐67 composites were elucidated through Monte Carlo simulations, modified attachment energy theory, and molecular dynamics simulations. This green one‐pot strategy offers a versatile platform for applications in controlled release, antimicrobial systems, and targeted delivery.

## Graphical abstract

Zeolitic imidazolate framework‐67 (ZIF‐67) was successfully synthesized in deionized water for 2 h in this work; meanwhile, various active substances could be encapsulated in one‐pot with the same process, resulting in high loading rates. The study delved into the growth mechanism, morphological difference and loading capacity. Additionally, the in vitro synergistic antibacterial performance of AS@ZIF‐67 was verified due to its pH‐responsive release performance.

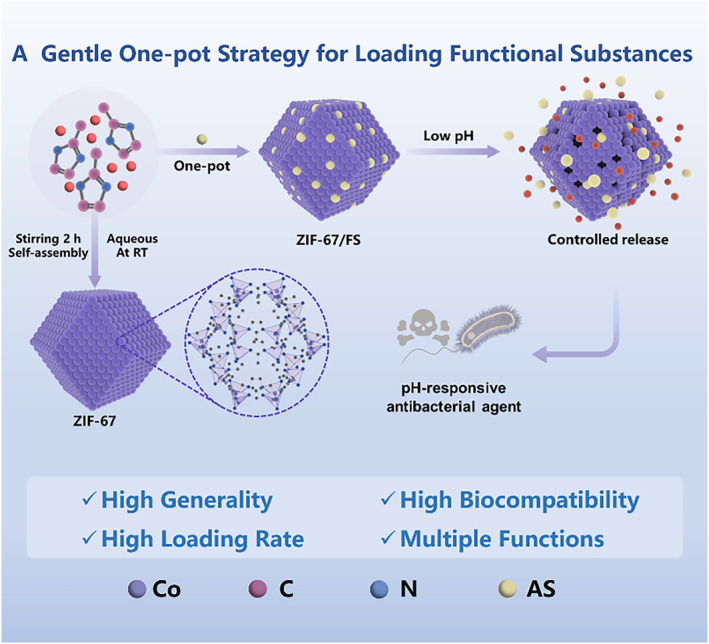

## 1 INTRODUCTION

Active substances (AS), including fragrances,[^1^, ^2^] polyphenols,^[3]^ proteins,[^4^, ^5^] vitamins,^[6]^ and pharmaceuticals, often suffer from inherent chemical instability and poor bioavailability,^[7]^ limiting their practical utility. Currently, an array of polymers,^[8]^ lipids,[^9^, ^10^, ^11^] and inorganic nanomaterials^[12]^ have been developed as carriers, with the aim of improving the stability and solubility of encapsulated AS, as well as prolonging circulation or retention times to augment safety and efficacy.^[13]^ While most researchers have hitherto focused on the delivery of single and specific substances, there have been comparatively few studies exploring general delivery systems capable of accommodating the diverse range of active components mentioned above. The development of such versatile delivery modalities can provide possibilities for the future collaborative application of multiple active components across a plurality of domains. Therefore, the conceptualization and materialization of general delivery platforms for these AS needs to be put on the agenda.

Among the various reported materials, metal‐organic frameworks (MOFs) feature large specific surface area,^[14]^ high porosity,^[15]^ adjustable pore size,[^15^, ^16^] and satisfactory loading capacities, rendering them capable of efficaciously delivering functional payloads.^[17]^ In particular, the zeolitic imidazolate frameworks (ZIFs), composed of imidazolate ligands and transition metal cations, possess a well‐defined structure and tunable morphology and constitutes one of the best delivery carriers.^[18]^ While the zinc‐based ZIF‐8 has been extensively studied for drug delivery due to its good biocompatibility and pH‐responsive degradation, the cobalt‐based ZIF‐67 presents stronger metal‐ligand coordination, enhanced thermal and chemical stability, and unique catalytic properties arising from cobalt centers.[^19^, ^20^, ^21^, ^22^] Notably, the cobalt ions in ZIF‐67 can participate in Fenton‐like reactions, generating reactive oxygen species for antibacterial and antitumor applications‐a feature absent in ZIF‐8. These characteristics make ZIF‐67 particularly suitable for applications requiring both delivery functionality and additional therapeutic effects. So far, most studies have revolved around the preparation of the AS‐loaded ZIF‐67 via post‐synthetic impregnation techniques. However, this post‐loading method typically engenders suboptimal encapsulation efficiencies, while also entailing a cumbersome preparative process.[^23^, ^24^] In contrast, the one‐pot, in situ encapsulation strategy not only dramatically improves the loading rate (LR) but also relatively avoids the risk of leakage of AS, relative to the conventional post‐loading approaches.

Hitherto, a multiplicity of synthesis methods for ZIF‐67 have been developed, including hydrothermal,^[25]^ solvothermal,^[26]^ and microwave‐assisted approaches.^[27]^ In the majority of these synthetic protocols, the addition of a deprotonated solvent has been a ubiquitous feature, with methanol^[28]^ and ammonium hydroxide^[29]^ being two commonly used organic solvents. However, the prevalent use of such solvents not only escalates manufacturing costs but also poses nonnegligible safety and environmental risks, thereby significantly hindering the industrial and commercial viability of ZIF‐67 applications from both environmental and economic standpoints. Conversely, although water represents a cheap, readily available and pollution‐free medium, the absence of another deprotonated solvent often leads to synthetic failure or the generation of poorly crystalline products. In view of this, some researchers have advocated for deprotonation through the imposition of prolonged reaction times, elevated temperatures and pressures. A typical example is that Qian et al.^[30]^ synthesized ZIF‐67 crystals within an aqueous solution for the first time, maintaining a ratio of Co^2+^: Hmim: H_2_O = 1: 58: 1100 and stirring the reaction mixtures at room temperature for 6 h. Similarly, Guo et al.^[25]^ subjected the ZIF‐67 precursors to an aqueous medium at 120°C for 30 min. In light of the significant issues of environmental pollution and laboratory safety, and with a commitment to green and safe practices, there is a pressing need to develop more moderate and environmentally friendly reaction conditions for the synthesis of ZIF‐67.^[31]^

**SCHEME 1:**
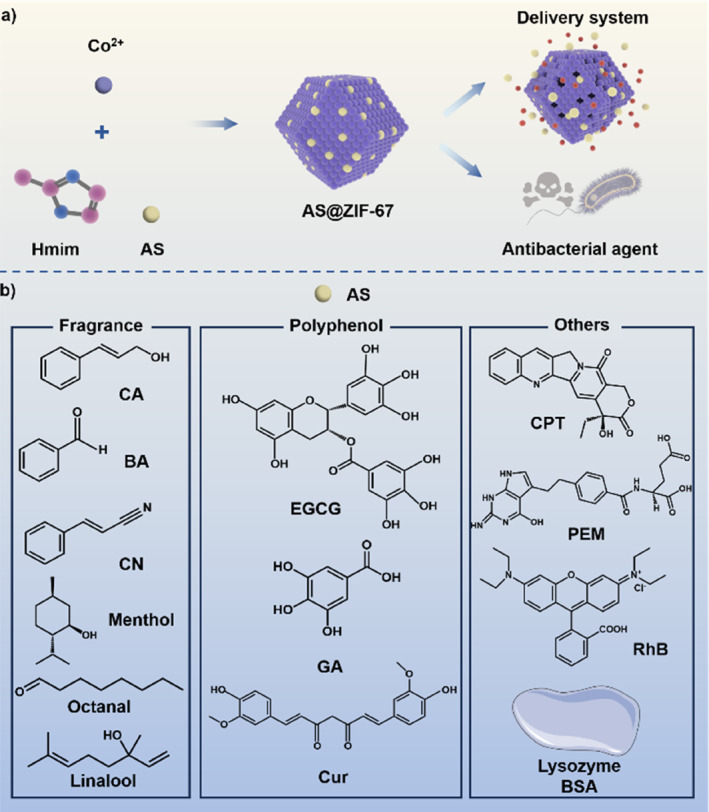
Schematic diagram of (a) the synthesis of AS@ZIF‐67 and (b) the structure of AS involved in this work. AS, active substances.

A versatile, pH‐responsive ZIF‐67‐based delivery system was developed via a facile one‐pot synthesis for the encapsulation and controlled release of diverse natural functionalities (Scheme 1). This methodology provided a green and efficient strategy to incorporate various agents, from small‐molecule fragrances to macromolecular enzymes, under mild aqueous conditions. Furthermore, molecular simulation methods were employed to elucidate the growth mechanisms and morphological evolution of the resulting nanoparticles. This study thus provided molecular‐level insights into the design of functional MOFs‐based carriers, offering a feasible approach for guiding the optimization and modification of MOFs materials.

## 2 MATERIALS AND METHODS

### 2.1 Synthesis of ZIF‐67 and functional substances loaded nanoparticles (AS@ZIF‐67)

Sixteen milligrams Co(NO_3_)_2_·6H_2_O was dissolved in 2 mL of deionized (DI) water. Then, 256.9 mg Hmim was dissolved in 2 mL of DI water. Those two solutions were mixed (Co^2+^: Hmim: H_2_O = 1: 58: 4000) and stirred at 500 rpm for 2 h at room temperature. The resulting purple precipitates were collected by centrifugation (12,000 rpm, 5 min), washed with DI water for 3 times, and finally vacuum‐dried at 50°C for 12 h. The other AS were encapsulated into ZIF‐67 through one‐pot synthesis, respectively (Scheme 1). AS@ZIF‐67 was prepared as mentioned above except for the addition of the corresponding AS with the desired amount (2 mg). Powder samples obtained via drying were defined as CA@ZIF‐67, BA@ZIF‐67, CN@ZIF‐67, EGCG@ZIF‐6, GA@ZIF‐67, Cur@ZIF‐67 and so on.

### 2.2 LR of AS@ZIF‐67

The AS LR of the system, that is, the mass fraction of AS, was calculated by the following Equation (1)^[32]^:

(1) $$\text{LR}\;\left(\%\right)=\frac{m_{\text{loaded}\;\text{AS}}}{m_{\text{AS}@\text{ZIF}‐67}}\times 100\%=\frac{m_{\text{added}\;\text{AS}}-m_{\text{AS}\;\text{in}\;\text{supernatant}}}{m_{\text{AS}@\text{ZIF}‐67}}\times 100\%,$$

where *m*_loaded AS_ is the mass of AS loading in the ZIF‐67, *m*_AS@ZIF‐67_ is the mass of AS@ZIF‐67 after drying, *m*_added AS_ is the mass of material put into the reaction, and *m*_AS in supernatant_ is the total mass of AS in the supernatant after centrifugation and washing. All mass was determined by ultraviolet‐visible (UV‐vis) spectroscopy at the respective maximum absorption peak, which corresponded to the standard curve.

### 2.3 Release profile

AS release from AS@ZIF‐67 was measured using a dialysis method at different pH values (5.5 and 7.4). Firstly, 10 mg AS@ZIF‐67 dissolved in 3 mL phosphate buffer saline (PBS) (10% ethanol [EtOH], pH 5.5 or 7.4) was put in a dialysis bag (M.W. 1000), and the dialysis bag containing the sample was immersed in 47 mL of PBS under shaking with a frequency of 150 rpm. At predetermined time intervals, 3 mL of dissolution sample was collected and an equal volume of fresh solution was added. The cumulative release rate of AS was determined by UV‐vis measurement at their respective maximum absorption wavelengths: cinnamyl alcohol (CA) (258 nm), benzaldehyde (BA) (250 nm), CN (410 nm), epigallocatechin gallate (EGCG) (273 nm), gallic acid (GA) (271 nm) and Cur (425 nm). The release percentage of AS was calculated using Equation (2):

(2) $$\text{Release}\;\left(\%\right)=\frac{m_{\text{released}\;\text{AS}}}{m_{\text{loaded}\;\text{AS}}}\times 100\%,$$

where *m*_released AS_ is the cumulative amount of AS released from AS@ZIF‐67.

### 2.4 Releasing kinetics

The AS release profiles were fitted using various mathematical models including Equation (3) zero‐order, Equation (4) first‐order, Equation (5) Higuchi, and Equation (6) Korsmeyer‐Peppas equation[^33^, ^34^, ^35^]:

(3) $$\frac{M_{t}}{M_{\infty }}=kt,$$

(4) $$\frac{M_{t}}{M_{\infty }}=a-e^{-\mathrm{kt}},$$

(5) $$\frac{M_{t}}{M_{\infty }}=kt^{0.5},$$

(6) $$\frac{M_{t}}{M_{\infty }}=kt^{n},$$

where *M*_*t*_/*M*_∞_ is the fraction of AS released at time *t* to the total content, and *k* is the release rate constant. The value of *n* is the release exponent. If the diffusion exponent (*n*) value is less than or equal to 0.45, drug release corresponds to Case I or the Fickian diffusion mechanism, 0.45 < *n* < 0.89 for anomalous or non‐Fickian transport, *n* = 0.89 for Case II transport, and *n* > 0.89 for super case II transport.^[36]^

### 2.5 Antibacterial assay

Luria–Bertani broth and LB agar were prepared according to the formula for bacterial culture. *Escherichia coli* and *Staphylococcus aureus* were used as Gram‐negative and Gram‐positive bacterial strains for the antibacterial research. A mono‐colony of *E. coli* or *S. aureus* formed in the solid medium was inoculated in liquid LB medium shaking with 100 rpm (37°C, 12 h). Then the antibacterial activity of AS, ZIF‐67, and AS@ZIF‐67 was evaluated by the Standard plate counting method and antibacterial ratio. *Escherichia coli* or *S. aureus* bacterial suspensions and H_2_O_2_ (100 μM), AS, ZIF‐67 (40 μg·mL^−1^, 100 μM H_2_O_2_), AS@ZIF‐67 (40 μg·mL^−1^, 100 μM H_2_O_2_) were mixed at pH 5.5 or 7.4 and incubated on a shaking plate for 12 h (100 rpm, 37°C). 50 μL of the mixture was then removed, diluted by 1000 in PBS, and coated in solid LB medium, before co‐incubation for 12 h (37°C). The bacterial survival rate was calculated by the number of *E. coli* and *S. aureus* colonies after incubation using Equation (7)^[23]^:

(7) $$\text{Bacterial}\;\text{survival}\;\text{rate}\;\left(\%\right)=\frac{{\text{CFU}}_{\text{experiments}\;\text{group}}}{{\text{CFU}}_{\text{control}\;\text{group}}}\times 100\%.$$

### 2.6 ·OH generation by CA@ZIF‐67 mediated Fenton‐like reaction

In the 1 M PBS (10% EtOH, pH 5.5 or 7.4, 4 mL), 1 mg/mL methylene blue (MB) solution (40 μL), 1 M H_2_O_2_ (20 μL) and CA@ZIF‐67 solution (the final concentration was 100 μg/mL) were mixed together. After incubation at 37°C for 2 h, the solutions were centrifuged and the absorbance was measured by UV‐vis spectroscopy.

### 2.7 In vitro cytotoxicity assay

For cell viability measurements, human normal hepatocytes L02 cells were plated in 96‐well plates at a density of 6 × 10^3^ cells per well and incubated for 24 h. The cells were then treated with samples (ZIF‐67, CA@ZIF‐67, and CA) at various concentrations in fresh RPMI 1640 medium containing 10% Fetal Bovine Serum (FBS). After 12 h of incubation, the medium was removed, and the cells were washed three times with PBS. Then, 100 μL of RPMI 1640 medium (without FBS) containing 10% CCK‐8 reagent was added to each well. Following 2 h of incubation at 37°C in a 5% CO_2_ atmosphere, the absorbance at 450 nm was measured using a microplate reader (SPARK 10M, TECAN). The experiment was performed in triplicate. Cell viability was calculated as follows Equation (8):

(8) $$\text{Cell}\;\text{viability}=\frac{\text{mean}\;\text{absorbance}\;\text{of}\;\text{test}\;\text{wells}-\text{mean}\;\text{absorbance}\;\text{of}\;\text{medium}\;\text{control}\;\text{wells}}{\text{mean}\;\text{absorbance}\;\text{of}\;\text{untreated}\;\text{wells}-\text{mean}\;\text{absorbance}\;\text{of}\;\text{medium}\;\text{control}\;\text{wells}\;}\times 100\%.$$

## 3 RESULTS AND DISCUSSION

### 3.1 Growth process and characterizations of ZIF‐67

**FIGURE 1:**
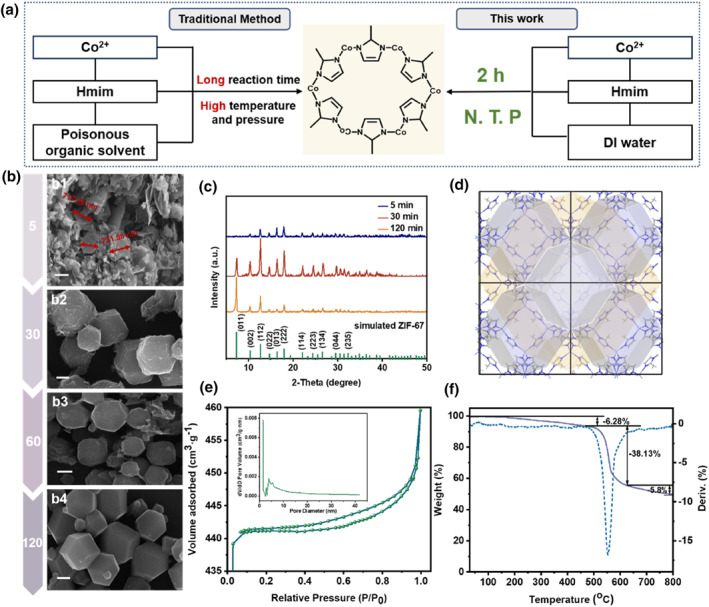
(a) The difference between the traditional method of ZIF‐67 and the synthesis proposed under normal temperature and pressure (N. T. P.) in this work. (b) Ex situ SEM analysis of ZIF‐67 grown for (b1) 5, (b2) 30, (b3) 60, and (b4) 120 min. Scale bar: 500 nm. (c) XRD patterns of ZIF‐67 at different time points and standard card. (d) Overlay of the crystal morphology (yellow) and the cage‐like structures (blue) in four crystal cells of ZIF‐67 (Note that it’s not the actual size ratio). (e) N_2_ sorption isotherms and BJH pore size distribution curve of ZIF‐67. (f) TGA‐DSC curves of ZIF‐67 in N_2_ atmosphere. BJH, Barrett–Joyner–Halenda; SEM, scanning electron microscope; TGA–DSC, Thermogravimetry–Differential Scanning Calorimetry; XRD, X‐ray diffraction; ZIF‐67, zeolitic imidazolate framework‐67.

In contrast to conventional ZIF‐67 synthesis methods that typically employ toxic organic solvents, prolonged reaction times, and elevated temperature/pressure conditions, this work focused on developing an efficient, rapid, and environmentally friendly synthetic approach. Specifically, DI water served as the sole solvent for ZIF‐67 synthesis under normal temperature and pressure conditions (Figure 1a). In order to gauge the underlying growth mechanism involving the formation of ZIF‐67 within this aqueous milieu, we conducted a series of experiments by tuning the time of coordination reaction, and recorded the corresponding scanning electron microscope (SEM) images and powder X‐ray diffraction (XRD) patterns of each stage. The distinctive growth process from amorphous nanosheets to dodecahedral crystal particles was successfully captured. In the initial 5‐min stage, the products consisted of unshaped nanosheets with an average diameter of 700 nm (Figure 1b1), exhibiting diminished crystallinity as evidenced by the weak (011) diffraction peak at 7.4° in the XRD pattern. Upon prolonging the reaction time to 30 min, the morphology transitioned to become uniform yet rough‐surfaced particulate structures of approximately 1400 nm (Figure 1b2), with strengthening of the (112), (222), and (011) diffraction signals. As the reaction progressed to 60 min, the surface flakes had completely dissipated (Figure 1b3). It was noted that perfect and uniform rhombic dodecahedron structures with an average size of 1682.74 ± 74.01 nm by SEM images were ultimately obtained after a 120 min treatment (Figure 1b4 and Figure S1a),^[37]^ which was in line with the size distribution with 1787.30 nm detected by dynamic light scattering (Figure S1b). The XRD pattern of the final samples matched well with the standard reference of ZIF‐67,^[38]^ confirming the successful synthesis of ZIF‐67 (Figure 1c). Extending the reaction time further to 240 min induced no significant changes about the size and structure, revealing the completion of the synthetic process (Figure S2). Based on the morphological evolution and XRD characterization, the optimized experimental condition was decided to be a 120‐min reaction duration.

**FIGURE 2:**
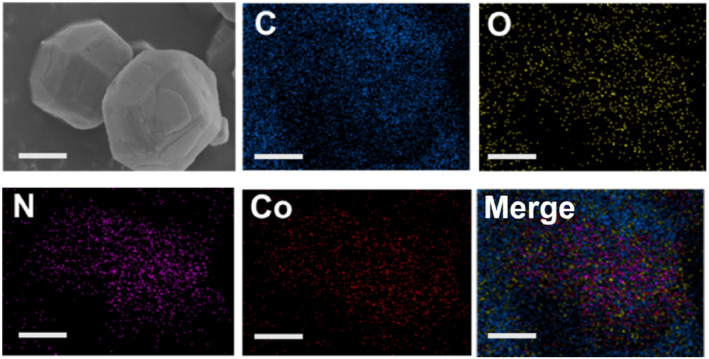
Elemental mapping images of zeolitic imidazolate framework‐67. Scale bar: 500 nm.

**FIGURE 3:**
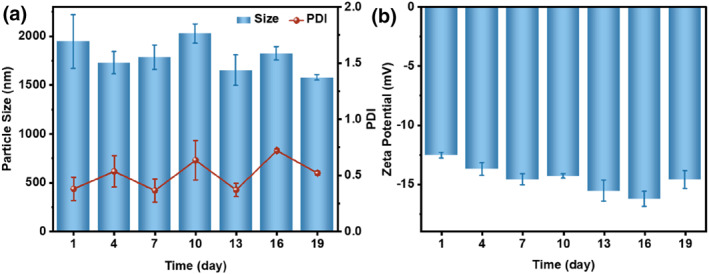
(a) Particle size, polymer dispersity index and (b) zeta potential of zeolitic imidazolate framework‐67 after 20 days of storage.

**FIGURE 4:**
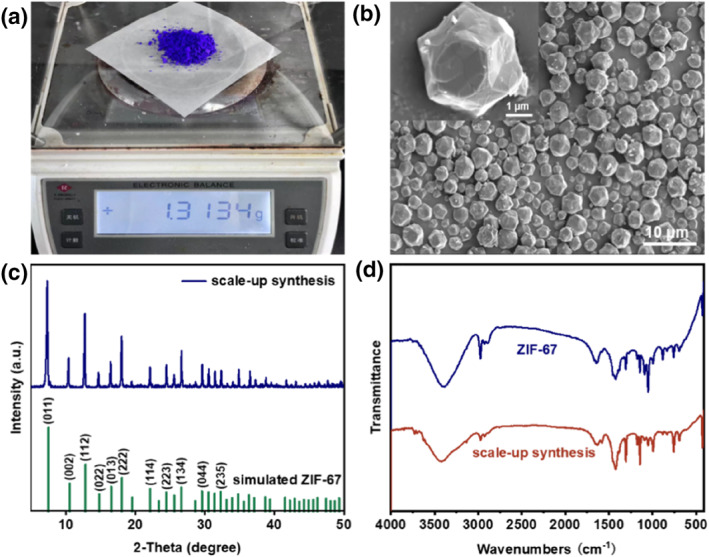
(a) 1313.4 mg ZIF‐67 attained in one batch in the scale‐up synthesis. (b) SEM, (c) XRD and (d) FT‐IR spectra of ZIF‐67 in the scale‐up synthesis. SEM, scanning electron microscope; XRD, X‐ray diffraction; ZIF‐67, zeolitic imidazolate framework‐67.

The attachment energy (AE) model^[39]^ is the basis used in this work to predict the crystal growth morphology based on the difference in crystal surface energy and the cell structure. The growth morphology directly determines the particle sizes and shapes of the equilibrium crystals.^[40]^ Therefore, we calculated the growth morphology of ZIF‐67, and the morphology prediction result was shown in yellow in Figure 1d. The growth morphology of ZIF‐67 was a dodecahedron composed of (110) group crystal planes with an aspect ratio of 1.414 and a symmetry multiplicity of 12, which is a rhombic ortho‐dodecahedron, which was consistent with our experimental results. As could also be seen, stacking with the crystal morphology and the cell structure was because of the constant repetition of cage‐like structures by which the three‐dimensional skeleton of ZIF‐67 was formed.^[41]^ According to the N_2_ adsorption‐desorption isotherms, the Brunauer‐Emmett‐Teller surface area of ZIF‐67 was 1289.35 m^2^g^−1^, which displayed a reversible type I isotherm^[42]^ (Figure 1e). Thermogravimetric analysis was subsequently conducted to evaluate the thermal stability of the ZIF‐67 framework. About 6.28% mass loss below 450°C possibly could be originated from residual water and unreacted ligands on the surface or in the pore of particles (Figure 1f).^[43]^ More obvious weight loss was observed above 450°C, likely owing to the destruction of ZIF‐67 skeleton.^[44]^ Therefore, almost constant weight before 450°C proved the high thermal stability of ZIF‐67, substantiating its viability for practical applications. The pore size was revealed to be 5.21 nm, consistent with previous reports (Table S1). As illustrated in the elemental mapping images (Figure 2), the distributions of C, O, N, and Co evidenced the formation of ZIF‐67. Furthermore, the colloidal stability of ZIF‐67 in PBS solutions was assessed, with no discernible changes in the hydrodynamic size (1788.84 ± 13.11 nm) or zeta potential (−14.54 ± 1.72 mV) even after 20 days of incubation (Figure 3), indicative of the excellent dispersity and long‐term storage ability of the particles. More importantly, the synthesis of ZIF‐67 had been successfully scaled up to gram scale in a laboratory setting, with the resultant material exhibiting comparable morphology, crystallinity, and structural properties to the small‐scale product (Figure 4).

### 3.2 Generality of the one‐pot synthesis of AS@ZIF‐67

To validate the universal encapsulation compatibility of ZIF‐67 in one‐pot synthesis, different types of functionalities were used to fabricate AS@ZIF‐67, including hydrophobic fragrant molecules, hydrophilic polyphenols, hydrophobic chemical drugs, fluorescent dyes, and hydrophilic macromolecule proteins. The experimental procedure closely resembled that of pure ZIF‐67 synthesis, except for the introduction of compounds mentioned above. A range of fragrances bearing distinct functional groups and odor types were employed to fabricate scented nanoparticles, such as spicy CA, green menthol, floral linalool with hydroxy group, as well as nutty BA, fruity octanal with aldehyde group, and spicy cinnamonitrile (CN) with cyan group. Moreover, EGCG, GA and curcumin (Cur) were utilized to investigate the effect of the numbers of phenolic hydroxy groups on the structure attributes of AS@ZIF‐67 nanoparticles. Additionally, considering the potential of ZIF‐67 in cancer therapy, particularly in chemodynamic therapy,^[44]^ clinically relevant hydrophobic drugs like pembrolizumab (PEM) and camptothecin were also incorporated to create a synergistic multifunctional nanoplatform for enhanced tumor therapy. Along with that, other dyes (rhodamine B, RhB) and macromolecule proteins (lysozyme and bovine serum albumin [BSA]) were included to further certify the universal applicability of the proposed one‐pot strategy.

**FIGURE 5:**
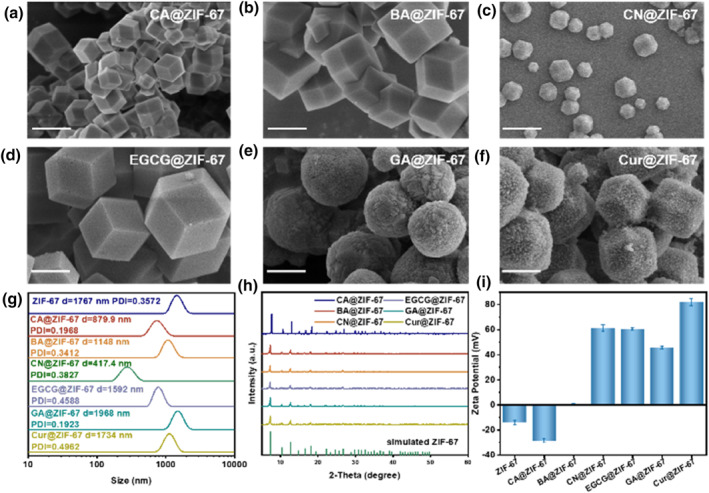
SEM images of (a) CA@ZIF‐67, (b) BA@ZIF‐67, (c) CN@ZIF‐67, (d) EGCG@ZIF‐67, (e) GA@ZIF‐67 and (f) Cur@ZIF‐67. Scale bar: 1 μm. (g) The DLS distribution, (h) XRD pattern and (i) zeta potential of pure ZIF‐67, CA@ZIF‐67, BA@ZIF‐67, CN@ZIF‐67, EGCG@ZIF‐67, GA@ZIF‐67 and Cur@ZIF‐67. SEM, scanning electron microscope; XRD, X‐ray diffraction; ZIF‐67, zeolitic imidazolate framework‐67.

**FIGURE 6:**
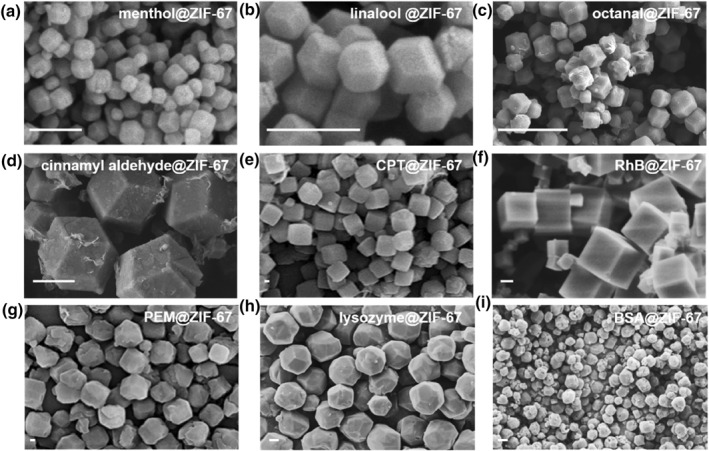
Ex situ SEM images of (a) menthol@ZIF‐67, (b) linalool@ZIF‐67, (c) octanal@ZIF‐67, (d) cinnamyl aldehyde@ZIF‐67, (e) CPT@ZIF‐67, (f) RhB@ZIF‐67, (g) PEM@ZIF‐67, (h) lysozyme@ZIF‐67 and (i) BSA@ZIF‐67. Scale bar: 1 μm. SEM, scanning electron microscope; ZIF‐67, zeolitic imidazolate framework‐67.

Obviously, the majority of AS@ZIF‐67 exhibited the characteristic polyhedron morphology, as shown in SEM images (Figures 5a–c and 6). However, a distinct granular morphology was observed for polyphenol@ZIF‐67 (Figure 5d–f), in which polyphenols could modulate crystal growth by affecting the coordination equilibria between metal ions and organic linkers, resulting in the formation of a spherical metal polyphenol network.[^45^, ^46^] Interestingly, CPT@ZIF‐67 and RhB@ZIF‐67 samples presented a cubic morphology, potentially owing to the strong interaction energy between AS and specific crystal facet, facilitating the growth of {100} crystal faces and the formation of cuboid crystals.^[47]^ When macromolecular proteins (lysozyme and BSA) were encapsulated, a more rounded surface with some fragmentation was observed, possibly indicating an incomplete reaction with the macromolecules. In addition to modulating the morphological features, this one‐pot approach also allows control over the particle size of the resulting AS@ZIF‐67 crystals (Figure 5g and Figure S3), which may be attributed to the varying deprotonation abilities of the AS.^[48]^ For instance, the fragrances containing cyan groups exhibited alkaline properties, making them more prone to deprotonate in the aqueous reaction medium. Within the ZIF‐67 formation system, the increased alkaline modulator content led to a higher pH, allowing more linker molecules in the deprotonated form to coordinate with metal ions and resulting in the reduced particle size of CN@ZIF‐67. These examples illustrate that by altering the pH of the reaction mixture with different alkaline compounds, MOFs of specific sizes can be readily produced.[^49^, ^50^] Subsequently, the relationship between molecular characteristics of AS and pore structure of ZIF‐67 was further investigated to underscore the loading and release mechanisms. The pore size of ZIF‐67 was determined to be 5.21 nm (Figure 1e), sufficiently large to accommodate most AS molecules used in this study (molecular dimensions <2 nm, Table S2). Hydrophobic molecules like CA and BA showed moderate loading efficiencies (12.17%–12.47%), while hydrophilic polyphenols like GA achieved exceptionally high loading (56.11%), suggesting that both molecular size and polarity influenced the encapsulation efficiency. These differences in loading behavior could be further attributed to the structural properties of ZIF‐67, particularly its large surface area of 1289.35 m^2^/g, which provided ample adsorption sites for diverse AS molecules.

Furthermore, the interaction energy (*E*_int_) between ZIF‐67 and different fragrant molecules was calculated by molecular dynamics simulation in order to compare the differences of crystal morphology according to the modified attachment energy (MAE) model.^[51]^ Molecular dynamics simulation is based on Newton's law of motion to solve the trajectory of each atom with time,^[52]^ which can simulate the actual motion process of molecules. The *E*_int_ calculated by the simulation results are summarized in Table 1, which represents the relative strength of the interactions between each CA/BA molecule and ZIF‐67. The number of added fragrance molecules in the simulation system was determined from the results of the adsorption simulations. The detailed calculation process could be found in Table S3. According to the MAE model, the larger the absolute value of *E*_int_, the slower the growth of the crystal face, leading to a larger crystal face area. It could be seen from Table 1 that the system with the highest *E*_int_ among CA@ZIF‐67 was CA2@ZIF‐67 and that among BA@ZIF‐67 was BA4@ZIF‐67. What is more, the *E*_int_ of BA4@ZIF‐67 was higher than that of CA2@ZIF‐67, which was consistent with the experimental results: The particle size of BA@ZIF‐67 was larger than that of CA@ZIF‐67 (Figure 5g).

**TABLE 1:** Interaction energy between ZIF‐67 and the fragrance molecules under different loadings.

CA@ZIF‐67		BA@ZIF‐67	
Systems	*E*_int_ (kJ/mol)	Systems	*E*_int_ (kJ/mol)
CA1^a^@ZIF‐67	−59.739	BA1@ZIF‐67	−72.924
CA2@ZIF‐67	−239.459	BA2@ZIF‐67	−285.363
CA3@ZIF‐67	−73.038	BA3@ZIF‐67	−191.326
/	/	BA4@ZIF‐67	−383.176
/	/	BA5@ZIF‐67	−248.864

Abbreviation: ZIF‐67, zeolitic imidazolate framework‐67.

^a^The number behind BA or CA represents the number of fragrance molecules added into a crystal cell of ZIF‐67.

In order to further explore the chemical properties of AS@ZIF‐67 following successful encapsulation, a selection of active compounds including CA, BA, CN, EGCG, GA and Cur were screened as representatives. The LR serves as a crucial parameter to assess delivery potential. Detailed calculations utilizing UV‐vis revealed the LR for CA@ZIF‐67, BA@ZIF‐67, CN@ZIF‐67, EGCG@ZIF‐67, GA@ZIF‐67 and Cur@ZIF‐67 to be 12.17%, 12.47%, 16.80%, 27.95%, 56.11% and 22.2%, respectively (Table S4; Figures S4 and S5). Meanwhile, UV‐vis spectra proved the existence of AS through absorption peaks of AS or ligand‐to‐metal charge transfer bonds (Figure S6).[^13^, ^53^]

**FIGURE 7:**
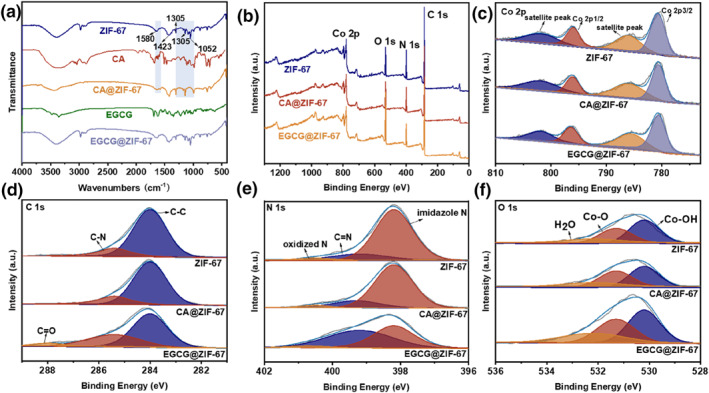
(a) FT‐IR spectra of ZIF‐67, CA, CA@ZIF‐67, EGCG and EGCG@ZIF‐67. (b) XPS, (c) high resolution Co 2p, (d) C 1s, (e) N 1s and (f) O 1s spectra of ZIF‐67, CA@ZIF‐67 and EGCG@ZIF‐67. CA, cinnamyl alcohol; EGCG, epigallocatechin gallate; XPS, X‐ray photoelectron spectroscopy; ZIF‐67, zeolitic imidazolate framework‐67.

**FIGURE 8:**
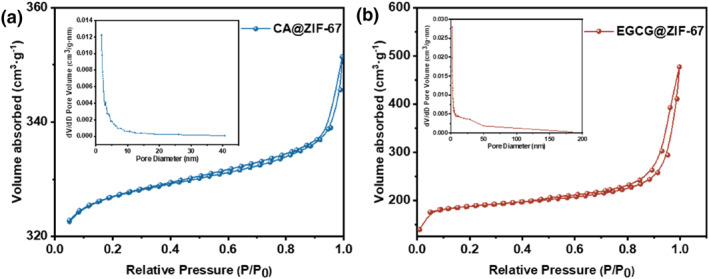
N_2_ sorption isotherms and BJH pore size distributions of (a) CA@ZIF‐67 and (b) EGCG@ZIF‐67.

XRD patterns of AS@ZIF‐67 corresponded well with the characteristic peaks of the simulated ZIF‐67 (Figure 5h), suggestive of the crystal structures of synthetic AS@ZIF‐67. As exhibited in Figure 5i, the zeta potential of ZIF‐67 was measured to be −14.41 ± 1.76 mV, while the zeta potential of AS@ZIF‐67 ranged from −28.99 to 81.82 mV due to the incorporation of different functional groups from AS, confirming the successful encapsulation of AS within the ZIF‐67 framework. The absorption peak at approximately 1580 cm^−1^ in Fourier Transform Infrared Spectroscopy (FT‐IR spectra) corresponded to the stretching vibration of C=N in Hmim,^[54]^ while the absorption peak within the range of 1350 to 1500 cm^−1^ was attributed to the stretching vibration of the imidazole ring. The absorption peak observed between 900 and 1350 cm^−1^ could be assigned to both the imidazole ring and out‐of‐plane bending vibrations (Figure 7a).^[55]^ This hypothesis was substantiated through X‐ray photoelectron spectroscopy (XPS) analysis (Figure 7b), where the Co 2p XPS spectra of ZIF‐67 exhibited peaks at 781.5 and 797.3 eV, corresponding to Co 2p 3/2 and Co 2p 1/2, respectively.^[56]^ Satellite peaks at binding energies of 786.6 and 803.1 eV were observed for Co atoms (Figure 7c), then by inference, the electronic structure remained unaltered following CA and EGCG loading. The C 1s spectrum exhibited peaks at 284.6 and 285.4 eV for C‐C and C‐N, but EGCG@ZIF‐67 had an extra peak at 289.1 eV for C=O, confirming successful encapsulation (Figure 7d). The N 1s spectrum could be fitted to three peaks at 398.7, 399.2, and 400.7 eV (Figure 7e), assigned to imidazole N, C‐N and oxidized N, dividedly. The increase of intensity of C = N in EGCG@ZIF‐67 was related with the decrease of intensity of imidazole N, demonstrating that the competitive coordination of polyphenols with metals resulted in a decrease in the amount of imidazole. The O 1s XPS spectra revealed peaks corresponding to Co‐OH, Co‐O, and H_2_O at 530.2, 531.3, and 532.2 eV, respectively, with an increased H_2_O peak area in EGCG@ZIF‐67 due to the hydrophilicity of EGCG (Figure 7f). N_2_ adsorption–desorption analysis revealed the classical type I isotherm curves of CA@ZIF‐67 and EGCG@ZIF‐67 with specific surface areas of 1342.13 and 729.88 m^2^·g^−1^ , with no significant variance in pore size distribution (Figure 8). We suspected that the reason why the specific surface areas of EGCG@ZIF‐67 narrowed down relatively was that metallic polyphenol nanoparticles encapsulated on the surface. The absence of discernible variance in pore size distribution could be attributed to the dense nature of ZIF‐67, resulting from pore blockage during its formation stage or a high skeleton density due to electrostatic or coordination action with AS.

### 3.3 Monte Carlo simulation of AS@ZIF‐67

To further investigate the adsorption and release of AS molecules in ZIF‐67 and the reason for the different particle sizes of AS@ZIF‐67 crystals due to the influence of encapsulated AS at the molecular level, molecular simulation methods were used to calculate the microscopic properties of different AS@ZIF‐67 systems. CA@ZIF‐67 and BA@ZIF‐67 were selected as a case for the comparison of simulation results.

**FIGURE 9:**
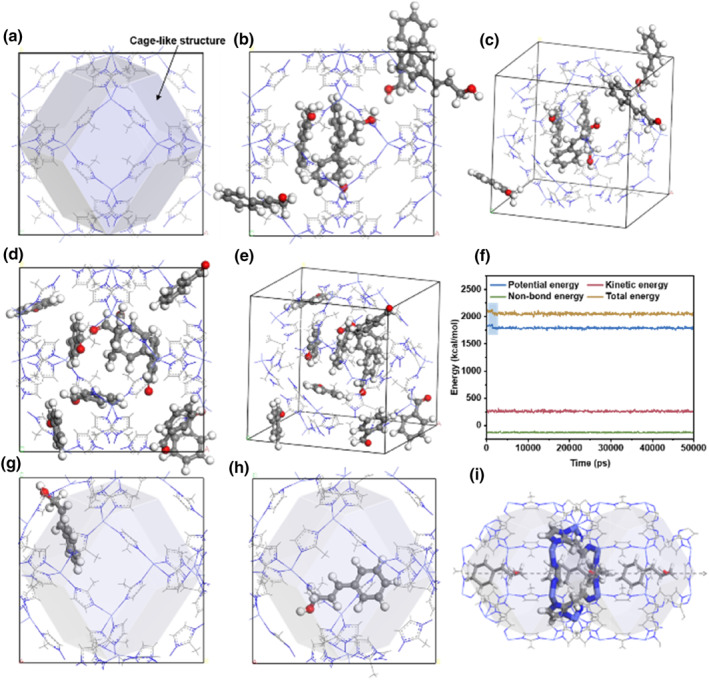
Crystal cell structures of (a) the standard ZIF‐67 crystal, (b and c) CA@ZIF‐67 simulation system, (d and e) BA@ZIF‐67 simulation system. (f) Energy variation of the CA@ZIF‐67 system with simulation time during the equilibrium dynamics simulation. (g and h) Initial and stable structure of CA@ZIF‐67 for the equilibrium dynamics simulation. (i) Specific process of the non‐equilibrium dynamics simulation.

Monte Carlo simulation is a numerical computational method based on probability and statistics theory, which is widely used in the field of molecular simulation.^[57]^ It estimates various thermodynamic or kinetic properties of a simulation system by randomly sampling molecular conformations and states. Adsorption simulations were first performed to determine the average loading of CA and BA in ZIF‐67. Figure 9a shows a single crystal cell structure of the standard ZIF‐67 crystal,^[41]^ which was modeled as a three‐dimensional periodic unit. Figure 9b,c revealed an example of the molecular lowest‐energy conformation of the CA@ZIF‐67 system during the simulation and Figure 9d,e demonstrated that of the BA@ZIF‐67 system. The results displayed that the average loading number of CA molecules in a crystal cell was 5.51 (739.28 g/(mol × cell)), while that of BA molecules was 8.93 (947.55 g/(mol × cell)). This fact agreed with the qualitative relationship of the two molecular LR determined experimentally (12.17% for CA and 12.47% for BA). The average isosteric heat of adsorption of a CA molecule was 24.02 kcal/mol while that of BA was 20.23 kcal/mol, which meant that compared to CA, BA was easier to adsorb or desorb. It could also be seen from Figure 9b–e that all the fragrant molecules were encapsulated in the “cage”‐like structure of ZIF‐67 after the simulation. Limited by the molecular interactions between the fragrance molecules and ZIF‐67, a cage‐like structure could be loaded with up to 3 CA molecules or 5 BA molecules. Therefore, the AS@ZIF‐67 systems with 1–3 CA molecules and 1–5 BA molecules were determined to be used for subsequent molecular dynamics simulations.

### 3.4 Molecular dynamics simulation of CA@ZIF‐67 and BA@ZIF‐67

In addition, we further modeled the dynamic process and its inverse process of AS molecules spanning the cage‐like structure of ZIF‐67, which was considered to be the diffusion and release behavior of AS in the ZIF‐67 crystal. To exclude any possible interference, only one CA molecule was placed in a crystal cell. The energy variation of the system with simulation time is shown in Figure 9f. It could be seen that there is a sudden change in the potential energy curve at about 2000 ps, which leads to the same change in the total energy curve. We first simulated the spontaneous entry process of the CA molecule into the cage and analyzed its stable loading state after entry, which was achieved by molecular dynamics simulation similar to that described above. The difference was that the CA molecule was placed at the boundary of two adjacent cage‐like structures this time (Figure 9g). This initial structure was used for the following simulation. Through the visual inspection of the simulation trajectories, we observed that the CA molecule entered the central cage from the boundary immediately at the beginning of the simulation and then remained stable in the cage (Figure 9h). This explained the sudden change in the energy curves. The same phenomenon was also observed in the simulation system loading with BA, suggesting the possibility of ZIF‐67 spontaneously loading AS.

The inverse process of the above was investigated by one of the non‐equilibrium molecular dynamics simulation methods, called steered molecular dynamics simulation.^[58]^ We applied an external force to the CA and BA molecules to pull them from one cage to another neighboring cage (Figure 9i). The atoms at the boundary of the two cages were set as the reference group for the pulling distance. The strength of the force was considered as the ability of the AS to release or dissociate from ZIF‐67. From Figure S7a and b, the required pulling force gradually increased as the fragrance molecules approached until they crossed the cage boundary. Then, a sudden change in the strength of the pulling force occurred at the moment when fragrance molecules overcame the intermolecular force and structural conflicts, thus spanning the boundary. Figure S7c and d shows the distance of the fragrance molecules relative to the boundary atoms during this process. The sudden change at the same time as the pulling force could also be observed, suggesting that fragrance molecules could be loaded stably in the cages of ZIF‐67 to some extent. What is more, the CA molecule required a higher maximum pulling force than the BA molecule, which meant that CA molecules were more stable while BA molecules were easier to release from ZIF‐67.

### 3.5 pH‐responsive release profiles of AS

Leveraging the stability ZIF‐67 in neutral environments and its decomposition under acidic conditions, we further studied the release of CA@ZIF‐67 and EGCG@ZIF‐67 in PBS solutions to assess their pH‐responsive delivery properties as nanocarriers. As depicted in Figure S8a and d, rapid and complete release of free CA and EGCG occurred within 6 h, with minimal dependence on the surrounding pH environment. In contrast, CA and EGCG encapsulated within ZIF‐67 exhibited sustained release patterns and demonstrated pH‐responsive behavior. Under acidic condition (pH 5.5), approximately 70% of the loaded CA was deliberated from CA@ZIF‐67 within 12 h, followed by a gradual increase in release rate until equilibrium at 48 h. Similarly, up to 37% of the encapsulated EGCG was released within 12 h, with this percentage increasing to 53% within 48 h. The accumulative release of both CA and EGCG decreased by approximately 10% in the simulated physiological environment. Subsequently, four pharmacokinetic models (zero‐order, first‐order, Higuchi and Korsmeyer–Peppas) were fitted to the experimentally derived CA and EGCG release profiles.^[33]^ The release profile of CA@ZIF‐67 showed a strong correlation with the first‐order model (*R* ^2^ > 0.99, Figure S8b and c), representing that CA release was primarily driven by the concentration gradient without external force. For EGCG@ZIF‐67, the Korsmeyer‐Peppas provided a good fit (*R* ^2^ > 0.98, Figure S8e,f), with a diffusion coefficient “*n*” ranging from 0.25 to 0.29 (which is <0.45), suggesting a Fickian diffusion mechanism.^[36]^ The breaking of coordination bonds might provide the impetus for diffusion and eruption of the Fickian mechanism. The other two models are shown in Figures S9 and S10.

### 3.6 Antibacterial performance

**FIGURE 10:**
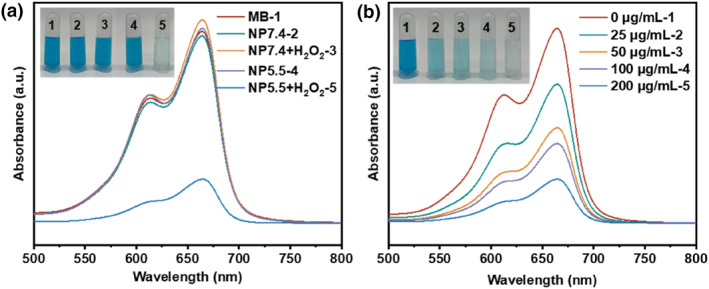
(a) UV‐vis absorption spectra and photograph (inset) of the PBS solutions (pH 7.4 or 5.5) containing MB, 1 M H_2_O_2_, and CA@ZIF‐67 NP. (b) UV‐vis absorption spectra of the PBS solutions (pH 5.5) containing MB, 1 M H_2_O_2_, and various concentrations of CA@ZIF‐67. MB, methylene blue; NP, nanoparticles; PBS, phosphate buffer saline; UV‐vis, ultraviolet‐visible.

The ·OH generating activity of AS@ZIF‐67‐induced Fenton‐like reaction was evaluated using the MB assay, which can be oxidized by the highly reactive OH into colorlessness accompanied with the decline of maximum absorbance at about 665 nm. As exhibited in Figure 10a, CA@ZIF‐67 alone had a negligible effect on the absorbance of MB even when the incubation duration was extended to 2 h. In contrast, the color fading of MB solution was observed and the absorbance intensity of MB significantly reduced after treatment with CA@ZIF‐67 and H_2_O_2_ in 1 M PBS (pH 5.5) solution together, validating the superior OH production capability. It was worthy to mention that the absorbance of MB had no detectable change with the same process in PBS solution (pH 7.4), which suggested the significance of acid environment in the AS@ZIF‐67‐mediated Fenton‐like reaction. Meanwhile, the variation in the concentration of nanoparticles had an obvious effect on the decomposition of MB (Figure 10b).

**FIGURE 11:**
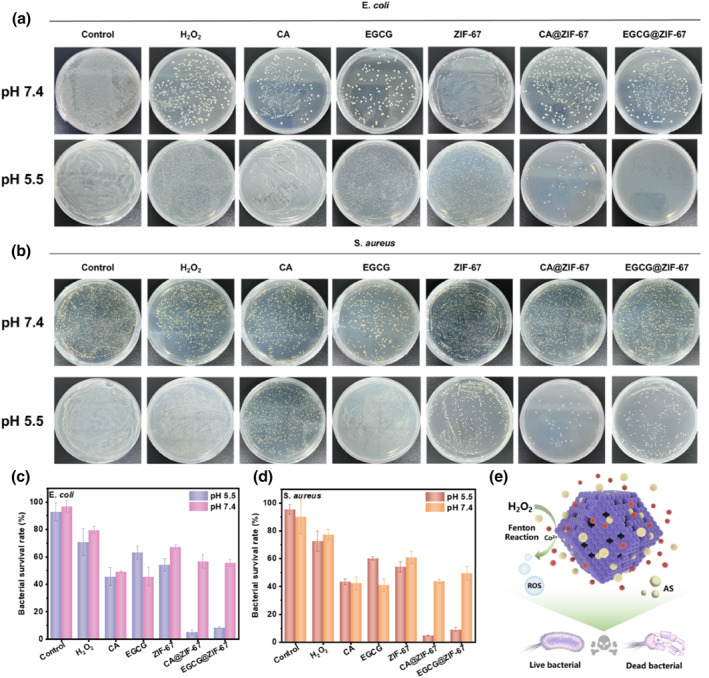
(a and b) Photographic images of bacterial colonies at different pH values (5.5 and 7.4). (c and d) Bacterial survival rate at different pH values (5.5 and 7.4). (e) Synergistic antimicrobial mechanism of AS@ZIF‐67.

Motivated by the excellent ·OH generating performance of ZIF‐67 along with the antibacterial activity of CA, thereafter, the antimicrobial property of CA@ZIF‐67 was further evaluated by the plate colony count method against *E. coli* and *S. aureus* in neutral and acidic environments. A large number of colonies survived on the agar plates inoculated with free CA and ZIF‐67, while few colonies appeared for the CA@ZIF‐67 group, demonstrating the synergistic antibacterial activity. Also, the results of the spread plate assay treated with EGCG@ZIF‐67 led to a similar conclusion (Figure 11a). Specifically, the decrease of bacteria number was greater than about 50% in comparison with free EGCG and ZIF‐67 groups (Figure 11c). Notably, both CA@ZIF‐67 and EGCG@ZIF‐67 showed obvious pH‐dependent bactericidal activity, with the *E. coli* inhibition ratio of 95.14% and 91.84% at pH 7.4, 43.49% and 44.63% at pH 5.5, respectively, which could be attributed to the effective release of Co^2+^ and CA under acidic condition, resulting in the highly toxic ·OH production and destruction of microbial cell membranes (Figure 11e).[^59^, ^60^, ^61^, ^62^, ^63^] Similar results were obtained on *S. aureus*, proving the excellent antibacterial capability of AS@ZIF‐67 (Figure 11b,d).

### 3.7 In vitro cytotoxicity assay

**FIGURE 12:**
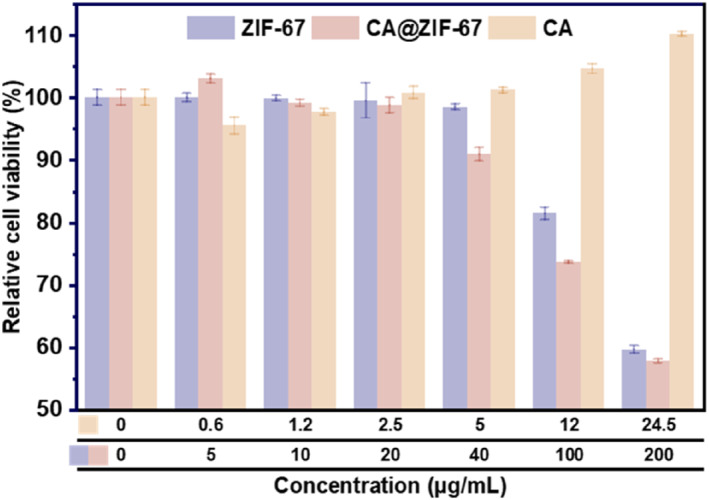
In vitro cell cytotoxicity of different concentrations of ZIF‐67, CA@ZIF‐67 and CA against L02 cells after 12 h incubation. CA, cinnamyl alcohol; ZIF‐67, zeolitic imidazolate framework‐67.

The cell viability of L02 cells after 12 h of treatment with ZIF‐67, CA@ZIF‐67, and free CA is shown in Figure 12. At low concentrations (<40 μg/mL), both ZIF‐67 and CA@ZIF‐67 maintained cell viability above 80%, indicating good biocompatibility. However, at higher concentrations, ZIF‐67 led to a decline in viability, which could be attributed to the release of Co^2+^ ions.[^23^, ^59^] Based on the results from the cell viability assay, both ZIF‐67 and CA@ZIF‐67 demonstrated minimal cytotoxic effects on L02 cells. Even at concentrations up to 200 μg/mL, the cell viability remained above 50%, indicating that these materials posed no substantial toxic risk to L02 cells within the tested concentration range. These findings suggest their favorable biocompatibility for potential biomedical applications.[^23^, ^45^]

## 4 CONCLUSION

In summary, we had developed a simple and gentle synthesis method of ZIF‐67 that could universally encapsulate AS in one pot, which included AS such as various hydrophobic molecules, hydrophilic molecules, and micro‐ and macromolecules. Firstly, we investigated the growth of ZIF‐67 during the synthesis process. Secondly, through a series of characterization, theoretical calculations, controlled release and in vitro antibacterial experiments, the multifunctionality and growth mechanism of AS@ZIF‐67 were discussed in detail. In addition, the molecular dynamics simulation results further revealed the properties and morphologies of different loaded AS molecules. This work opens up an entirely new approach for the multifunctional green synthesis of AS@ZIF‐67 and points the way to understanding and even improving the properties of related MOFs at the molecular level for use as promising transport carriers.

## CONFLICT OF INTEREST STATEMENT

The authors declare no conflicts of interest.

## ETHICS STATEMENT

This article does not contain any studies with human participants or animals performed by any of the authors.

## Supporting information

Supporting Information S1

## Data Availability

The crystal cell structure of the standard ZIF‐67 crystal was obtained from the CCDC database (https://www.ccdc.cam.ac.uk, Deposition Number: 671074), whose original source is from Banerjee et al.^[41]^ The molecular structures of CA and BA were obtained from the PubChem database (https://pubchem.ncbi.nlm.nih.gov).
