# Supplementary material for: A general and green approach of pH‐sensitive ZIF‐67 for encapsulating active substances
Source: Smart Mol. 2026 Mar 11;4(2):e70040. doi: 10.1002/smo2.70040 (PMC13317572; doi:10.1002/smo2.70040)
Supplement: Supplementary file 1 — Supporting Information S1 [file SMO2-4-e70040-s001.docx]

Supporting Information

**A General and Green Approach of pH-sensitive ZIF-67 for Encapsulating Active Substances**

Xinyi Sun^1^, Yan Qi^2^, Xinhao Che^2^, Yuxin Li^1^, Lulu Zhou^1^*, Lei Zhang^2^*, and Jing Hu^3^*

**Material and methods**

**1. Materials**

All chemicals were obtained from commercial suppliers and used without further purification. Cobalt nitrate hexahydrate [Co(NO_3_)_2_·6H_2_O], 2-methylimidazole (Hmim), ethanol (EtOH), cinnamyl alcohol (CA), benzaldehyde (BA), cinnamonitrile (CN), EGCG, gallic acid (GA), curcumin (Cur), phosphate-buffered saline (PBS), methylene blue (MB) were purchased from Titan Technology Co., ltd. 30% hydrogen peroxide (H_2_O_2_) were purchased from Sinopharm Reagent Co., ltd. The bacterial strains Staphylococcus aureus (S. *aureus*, ATCC 29213) and Escherichia coli (E. *coli*, ATCC 25922) were obtained from Shanghai Institute of Technology. Luria-Bertani (LB) broth powder and LB agar powder were purchased from BBI Life Sciences Co., ltd. Fetal bovine serum (FBS) was purchased from Gibco Invitrogen.

**2. Characterizations**

Scanning electron microscope (SEM) were collected on ZEISS GeminiSEM 300 (Germany) with an accelerating voltage of 3.00 kV. The hydrodynamic diameter, polydispersity index (PDI), and zeta potential of the sample particles were tested using a nanoparticle size potential analyzer (Zetasizer Pro, MalvemPanalytical, UK). The N_2_ adsorption-desorption isotherm and specific surface area were recorded by an automatic physical and chemical adsorption instrument (ASAP2460, Mac, USA) at 77 K. Powder X-ray diffration (XRD) patterns were recorded at room temperature on a Bruker D8 ADVANCE diffractometer (Germany). Ultraviolet-visible (UV-vis) absorption spectra were collected on a UV-vis spectrophotometer (Shimadzu UV-1900, Japan). Thermal gravimetric analysis (TGA) was measured on a thermal analysis instrument (Q5000IR, TA, USA) in a nitrogen flow. Fourier transform infrared spectroscopy (FT-IR) spectra were obtained using a Nicolet iN10 spectrometer (Thermo Fisher Scientific, Waltham, MA, USA). X-ray photoelectron spectroscopy (XPS) was performed with a Thermo ESCALAB 250XI multifunctional imaging electron spectrometer (Thermo Fisher Nexsa Scientific, USA). The specific surface area and pore volume of the products were determined by Brunauer-Emmett-Teller (BET) and Barett-Joyner-Halenda (BJH) methods (TriStar 3020, Micromeritics, America).

**3. Scale-up synthesis of ZIF-67**

To scale up the synthesis of ZIF-67, the same synthesis protocol was adopted as that used for ZIF-67. That is say, the reaction solution was prepared by mixing 200 mL Hmim solution (25.96g Hmim) and 200 mL Co(NO_3_)_2_·6H_2_O solution (1.6 g Co(NO_3_)_2_·6H_2_O). The remaining steps were the same as above.

**4. Stability study of ZIF-67**

The stability of ZIF-67 in terms of nanosuspension particle size, PDI and Zeta potential after 20 days at room temperature was evaluated. 20 mg ZIF-67 were dissolved in 50 mL PBS (pH 7.4), and took 3 mL every 3 day to test particle size, PDI and potential.

**5. Monte Carlo simulation**

The Monte Carlo simulations of fragrance adsorption in ZIF-67 were performed using Sorption Tools module in Materials Studio (MS) v2023. We first corrected the uncertainty of the hydrogen atoms in ZIF-67 due to the experimental determinations in order to import the structure correctly into MS software. Prior to the Monte Carlo simulations, geometric optimization was performed for ZIF-67, single CA molecule, and single BA molecule using Forcite module in MS. Sorption simulations were then performed at a fixed pressure of 1 atm and a temperature of 298K. Metropolis was selected as the simulation method and Universal force field in MS was selected for the simulation systems. The Ewald & Group method was selected for the calculation of electrostatic interactions and the Atom-Based method was selected for that of van der Waals interactions. Other parameters remained the default. Finally, 10 lowest-energy frames of the CA@ZIF-67 and BA@ZIF-67 systems were saved. The average loading and isosteric heat of CA and BA molecules were obtained from the simulation results. All the visualizations were performed in MS software.

**6. Modified attachment energy theory**

The growth morphology of ZIF-67 crystal was predicted according to the AE model using Morphology Calculation module in MS. Forcite was selected as the energy method during the simulations and the force field was also the universal. Other parameters remained the default.

The AE model can be simply described as Equation S1, where *E*_att_ is the attachment energy which represents the energy released on the attachment of a growth slice to a growing crystal face, *E*_latt_ is the lattice energy of the crystal, and *E*_slice_ is the energy of a growth slice of thickness *d*_hkl_. The relative growth rate (*R*_ij_) of the crystal face is proportional to its attachment energy, as shown in Equation S2. i.e., the face with a lower absolute value of attachment energy grows more slowly.

| $\text{E}_{\text{att}}\text{ = }\text{E}_{\text{latt}}\text{-}\text{ E}_{\text{slice}}$ | (S1) |
| --- | --- |
| $\text{R}_{\text{ij}}\text{ = }\frac{\text{R}_{\text{i}}}{\text{R}_{\text{j}}}\text{=}\frac{\text{ E}_{\text{att,i}}}{\text{E}_{\text{att,j}}}$ | (S2) |

The MAE model was used to analyze different particle sizes of AS@ZIF-67 systems. According to the MAE model, energy correction term *E*_s_ is introduced to correct the AE model. The MAE model can be simply described as Equation (S3), where *E*_m_ is the modified attachment energy of the crystal face and *E*_s_ is the environment-crystal interaction energy on the crystal face which is proportional to the total environment-crystal interaction energy *E*_int_. Therefore, the higher the absolute value of *E*_int_, the slower the growth of the crystal face, and thus the larger the crystal face area. In this paper, *E*_int_ was calculated according to Equation (S4), where *E*_total_ represents the total energy of a crystal cell structure, *E*_ZIF-67_ represents the energy of ZIF-67 in this crystal cell, and *E*_AS_ represents the total energy of all the AS molecules.

| $\text{E}_{\text{m}}\text{ = }{\text{(1-0.1×\vert}\text{E}_{\text{s}}\text{\vert)×E}}_{\text{att}}$ | (S3) |
| --- | --- |
| $\text{E}_{\text{int}}\text{ = }\text{E}_{\text{total}}\text{-}\text{E}_{\text{zif-67}}\text{-}\text{E}_{\text{AS}}$ | (S4) |

**7. Molecular dynamics simulation**

The molecular dynamics simulations of AS@ZIF-67 were performed by the GROMACS software.^1^ OPLS-AA force field^2^ was applied to describe the motion behavior of AS molecules involved in the simulations and the force field used for ZIF-67 was derived from Krokidas et al.^3^ Eight simulation systems were constructed according to the results of the sorption simulations. The number of CA molecules in ZIF-67 is from 1 to 3 and that of BA molecules is 1 to 5. These molecules were randomly filled into the cage of ZIF-67 without structural conflicts. 3D-periodic boundary conditions were applied to all the simulation systems. 50ns dynamics simulation was performed for each system in the canonical ensemble with the constant number of atoms (N), volume (V), and temperature (T, 298K) (NVT ensemble), and the simulation trajectories were saved every 100ps. The Lorentz-Berthelot combination rule was used for non-bonded interactions between AS molecules and ZIF-67, as well as between the atoms of neighboring periodic units. The cutoff value for electrostatic interactions was set to 6Å. The particle mesh Ewald (PME) method was used to calculate the remote electrostatic interactions. The covalent bonds involving hydrogen were constrained using the LINCS algorithm. All the above parameters were set in the GROMACS software.

The spontaneous entry of CA and BA molecules into the cage of ZIF-67 was simulated using a similar method to that described above. The difference is that the CA and BA molecules were placed at the boundary of two adjacent cages this time. Such structures were used as input for the molecular dynamics simulations. To exclude any possible interference, only one fragrance molecule was placed in a crystal cell.

Steered molecular dynamics simulation was also performed in GROMACS with an approach similar to that of Krokidas et al.^3^ We applied an external force to the CA and BA molecules to pull them from one cage to another neighboring cage. The reference group was determined by the mass center of the atoms at the junction of the two cages. The pulling direction was the line between the mass center of the AS molecule and the center of the “hole”, which in our system was (1, -1, 1). The simulation time was set slightly longer than the moment when the fragrance molecules crossed the boundary. The other simulation parameters remained the same as above.

**Table S1.** Reaction solvent, condition, surface area and pore characteristics parameters of ZIF-67 in different works.

| Different synthesis method of ZIF-67 | Solvent | Temperature | Time | S_BET_ (m^2^g^-1^) | Pore size (nm) | Ref. |
| --- | --- | --- | --- | --- | --- | --- |
| solvothermal synthesis | methanol | 120^º^C | 4 h | 1027 | 0.5 | 4 |
| methanol solution | methanol | RT | 26 h | 1994.69 | 1.2 | 5 |
| hydrothermal synthesis | water | 80^º^C | 24 h | 316 | 0.17 | 6 |
| aqueous synthesis | water | RT | 2 h | 1289.35 | 5.21 | This work |

**Table S2.** The molecular size of the AS.

| AS | Size (Å) | AS | Size (Å) |
| --- | --- | --- | --- |
| CA | 9.1 | EGCG | 10.3 |
| BA | 5.9 | GA | 7.6 |
| CN | 8.4 | Cur | 19.1 |
| Menthol | 7.9 | CPT | 13 |
| Octanal | 10.7 | PEM | 17 |
| Linalool | 8.9 | / | / |

**Table S3.** Calculation results of interaction energy between ZIF-67 and the fragrance molecules under different loading.

| CA1@ZIF-67 | | | | |
| --- | --- | --- | --- | --- |
| Energy (KJ/mol) | E_total_ | E_zif-67_ | E_AS_ | E_int_ |
| Vdw-SR | -214.197 | -202.086 | -0.857 | -11.254 |
| Coulomb-SR | -9720.540 | -9631.910 | -60.209 | -28.421 |
| Coulomb-LR | 2043.840 | 2014.390 | 49.514 | -20.064 |
| Total | -7890.897 | -7819.606 | -11.552 | -59.739 |
| CA2@ZIF-67 | | | | |
| Energy (KJ/mol) | E_total_ | E_zif-67_ | E_AS_ | E_int_ |
| Vdw-SR | -283.813 | -214.477 | -0.074 | -69.262 |
| Coulomb-SR | -9748.220 | -9565.630 | -114.878 | -67.712 |
| Coulomb-LR | 2109.650 | 2114.920 | 97.215 | -102.485 |
| Total | -7922.383 | -7665.187 | -17.737 | -239.459 |
| CA3@ZIF-67 | | | | |
| Energy (KJ/mol) | E_total_ | E_zif-67_ | E_AS_ | E_int_ |
| Vdw-SR | 154.986 | -176.210 | -5.957 | 337.154 |
| Coulomb-SR | -12364.100 | -9679.020 | -172.238 | -2512.840 |
| Coulomb-LR | 4363.220 | 2121.610 | 138.961 | 2102.649 |
| Total | -7845.894 | -7733.620 | -39.234 | -73.038 |
| BA1@ZIF-67 | | | | |
| Energy (KJ/mol) | E_total_ | E_zif-67_ | E_AS_ | E_int_ |
| Vdw-SR | 215.589 | 236.549 | -0.723 | -20.237 |
| Coulomb-SR | -12126.600 | -12065.100 | -61.642 | 0.142 |
| Coulomb-LR | 4302.360 | 4293.670 | 61.518 | -52.828 |
| Total | -7608.651 | -7534.881 | -0.846 | -72.924 |
| BA2@ZIF-67 | | | | |
| Energy (KJ/mol) | E_total_ | E_zif-67_ | E_AS_ | E_int_ |
| Vdw-SR | 204.765 | 236.382 | -1.259 | -30.358 |
| Coulomb-SR | -10008.200 | -12171.900 | -123.515 | 2287.215 |
| Coulomb-LR | 1950.400 | 4370.150 | 122.466 | -2542.220 |
| Total | -7853.035 | -7565.368 | -2.308 | -285.363 |
| BA3@ZIF-67 | | | | |
| Energy (KJ/mol) | E_total_ | E_zif-67_ | E_AS_ | E_int_ |
| Vdw-SR | 167.022 | 206.050 | -2.747 | -36.281 |
| Coulomb-SR | -12307.200 | -12113.200 | -182.093 | -11.907 |
| Coulomb-LR | 4339.860 | 4306.260 | 176.738 | -143.138 |
| Total | -7800.318 | -7600.890 | -8.102 | -191.326 |
| BA4@ZIF-67 | | | | |
| Energy (KJ/mol) | E_total_ | E_zif-67_ | E_AS_ | E_int_ |
| Vdw-SR | 135.299 | 187.707 | -5.267 | -47.141 |
| Coulomb-SR | -9971.310 | -12043.600 | -245.205 | 2317.495 |
| Coulomb-LR | 2006.910 | 4419.500 | 240.943 | -2653.530 |
| Total | -7829.101 | -7436.393 | -9.529 | -383.176 |
| BA5@ZIF-67 | | | | |
| Energy (KJ/mol) | E_total_ | E_zif-67_ | E_AS_ | E_int_ |
| Vdw-SR | 114.307 | 200.878 | -7.002 | -79.569 |
| Coulomb-SR | -8673.690 | -12104.900 | -303.965 | 3735.175 |
| Coulomb-LR | 816.237 | 4424.910 | 295.794 | -3904.470 |
| Total | -7743.146 | -7479.112 | -15.173 | -248.864 |

In Table S1, the energy of Vdw-SR represents Van der Waals interactions (short-range), the energy of Coulomb-SR represents the Coulomb interactions in real space (short-range), and the energy of Coulomb-LR represents the Coulomb interactions in reciprocal space (long-range). In GROMACS software, the calculation of interaction energy within a simulation system considers two kinds of intermolecular non-bond interactions, Van der Waals interactions and Coulomb interactions. Van der Waals interactions contain short-range Van der Waals interactions and long-range dispersion correction. Coulomb interactions contain short-range and long-range Coulomb interactions. Since the sizes of all our simulation systems do not exceed 16 Å, the dispersion correction term is ignored.

**Table S4.** The calculation process for LR of AS@ZIF-67.

| Substance name | Amount of the added AS (mg) | Absorbance of the diluted supernatant after reaction (a.u.) | Amount of the residual substance in the supernatant measured by UV-Vis (mg) | Amount of AS@ZIF-67 (mg) | Loading rate (%) |
| --- | --- | --- | --- | --- | --- |
| CA | 13.7 | 1.4860 | 0.158 | 19.9 | 12.17 ± 1.64 |
| BA | 13.4 | 0.2453 | 0.637 | 15.3 | 12.47 ± 2.34 |
| CN | 7.7 | 0.9874 | 0.589 | 20.3 | 16.80 ± 0.91 |
| EGCG | 16.0 | 0.1967 | 0.562 | 12.3 | 27.95 ± 1.33 |
| GA | 10.3 | 0.3595 | 0.521 | 6.2 | 56.11 ± 4.52 |
| CUR | 10.3 | 0.9506 | 0.227 | 17.0 | 22.20 ± 2.58 |


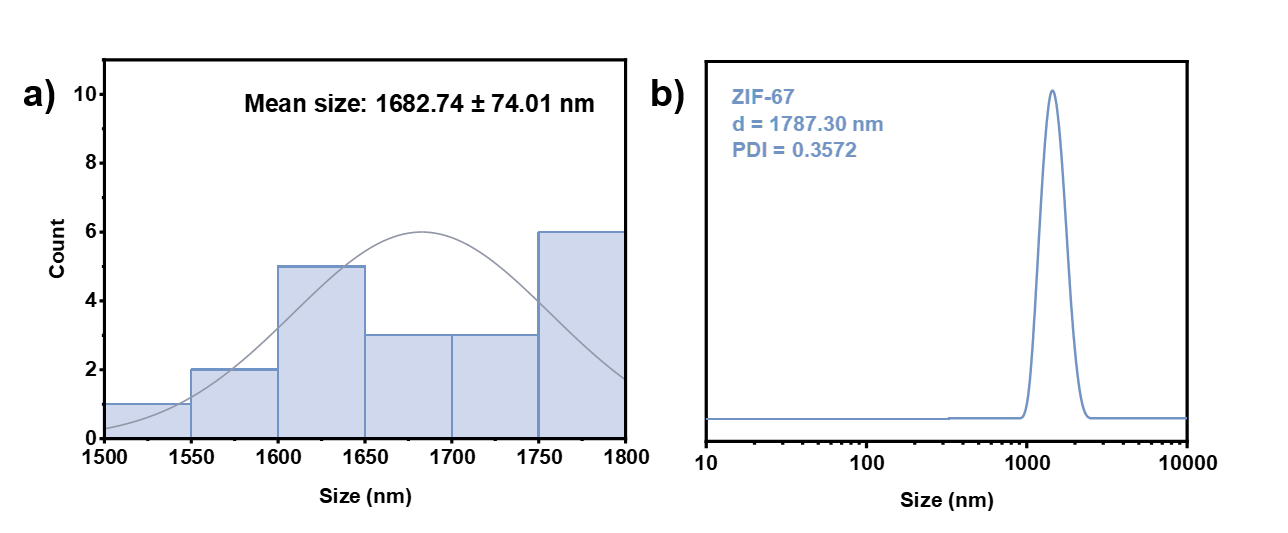


**Figure S1.** a) Corresponding size distribution histogram of ZIF-67. b) Hydrodynamic size of ZIF-67.


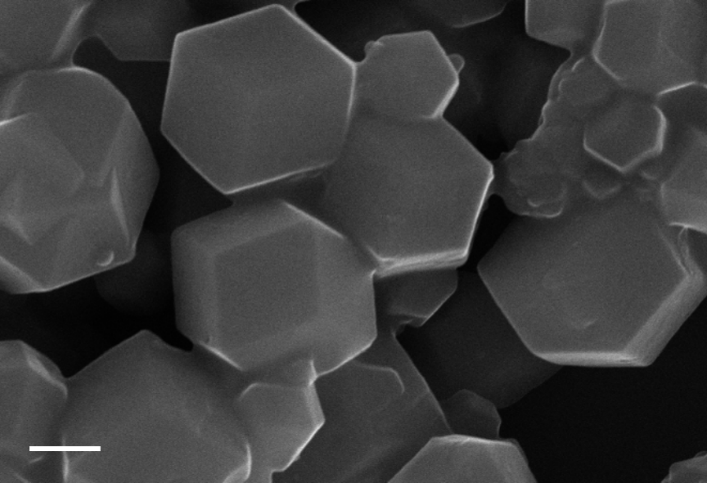


**Figure S2.** Ex situ SEM analysis of ZIF-67 growth for 240 min. Scale bar: 500 nm.


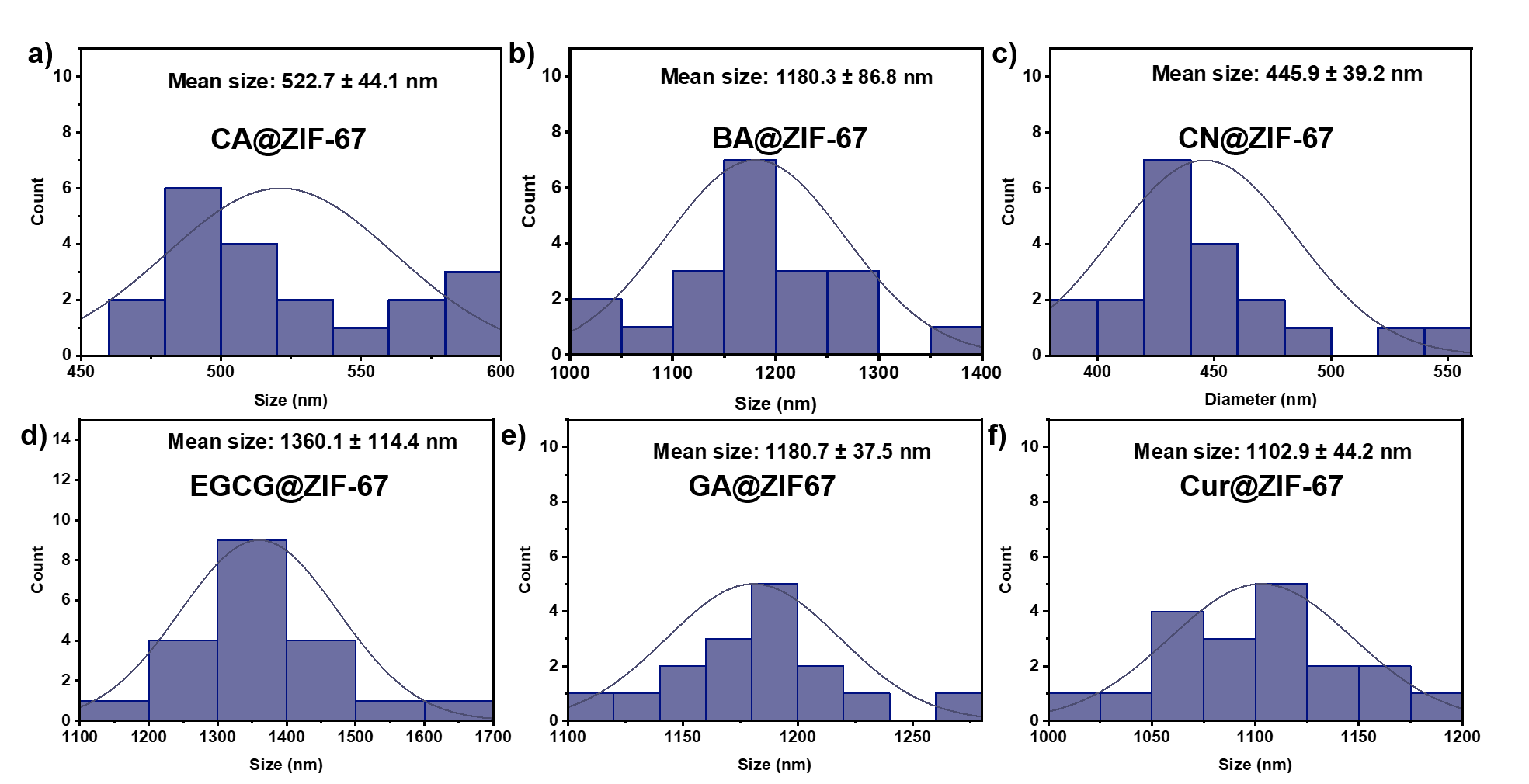


**Figure S3.** Corresponding size distribution histogram of a) CA@ZIF-67, b) BA@ZIF-67, c) CN@ZIF-67, d) EGCG@ZIF-67, e) GA@ZIF-67 and f) Cur@ZIF-67 from SEM images in Figure 5a-f, respectively.


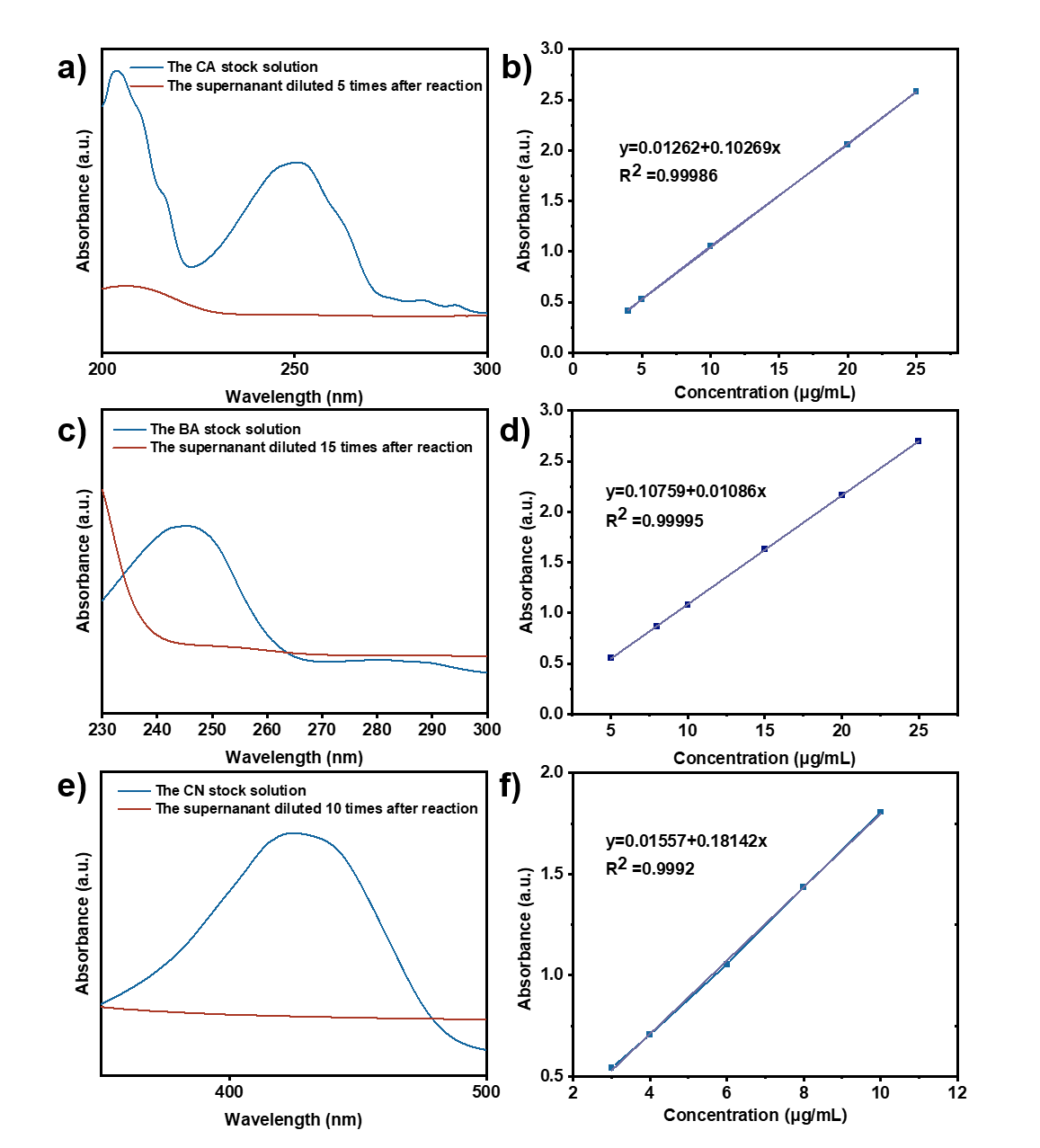


**Figure S4.** Quantification of CA, BA and CN loaded in the ZIF-67. a, c, e) The UV-vis spectra of the initial stock solution of CA, BA and CN and the final supernatant of AS after reaction. b, d, f) The Lambert-Beer law plot of the absorbance at characteristic peak with the regression equation.


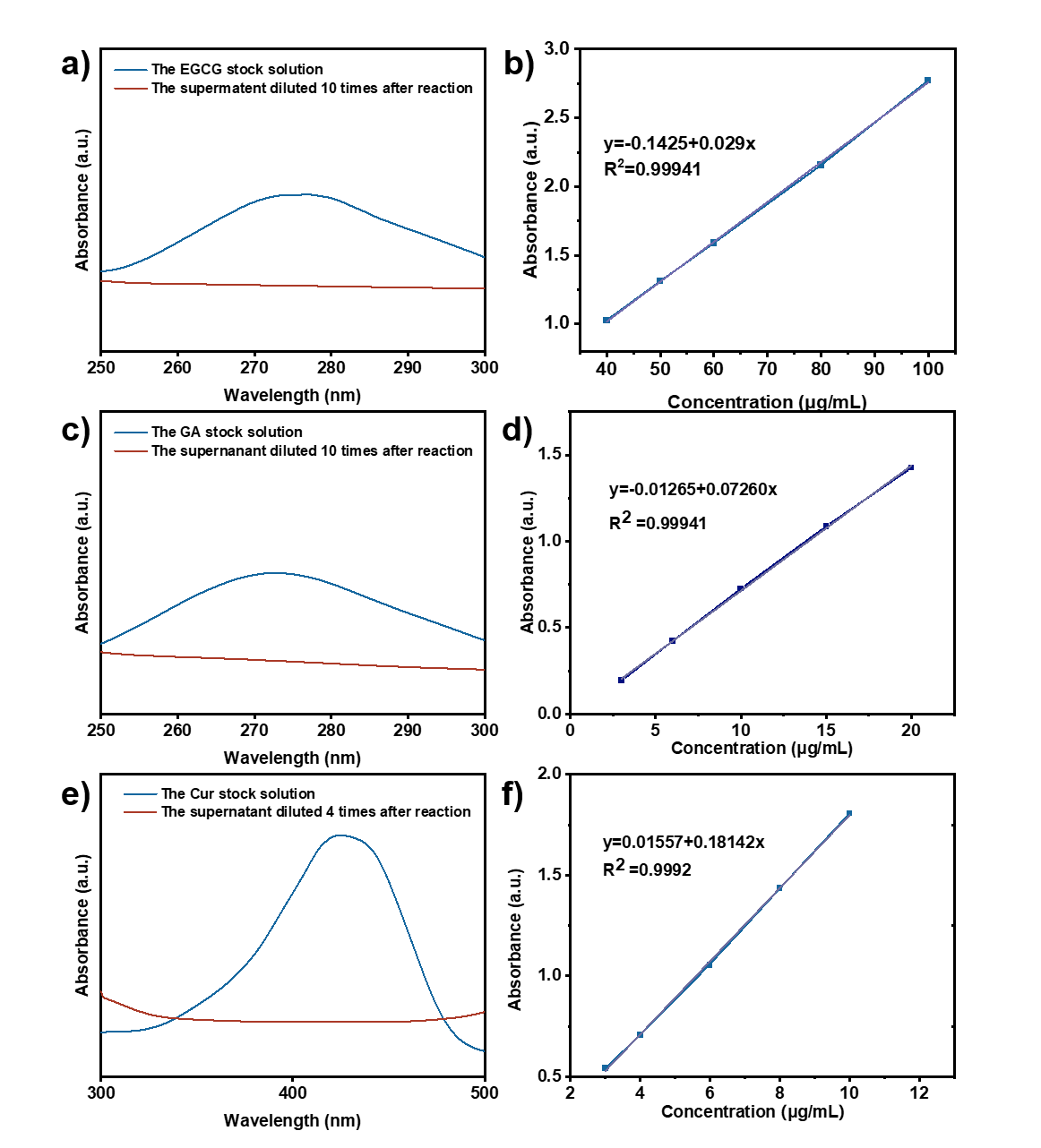


**Figure S5.** Quantification of EGCG, GA and Cur loaded in the ZIF-67. a, c, e) The UV-vis spectra of the initial stock solution of EGCG, GA and Cur and the final supernatant of AS after reaction. b, d, f) The Lambert-Beer law plot of the absorbance at characteristic peak with the regression equation.


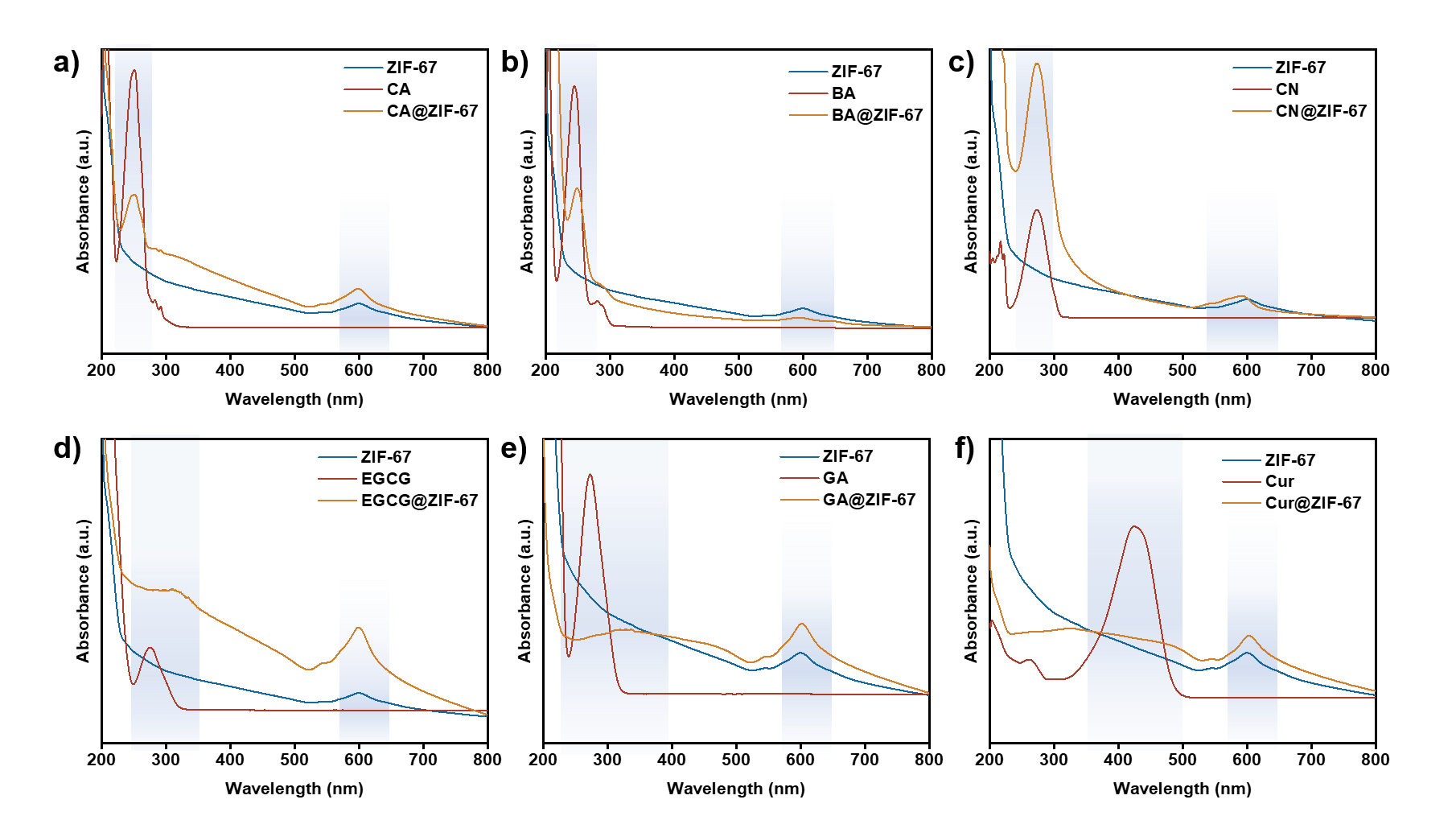


**Figure S6.** UV-vis spectra of free AS, ZIF-67 and AS@ZIF-67.


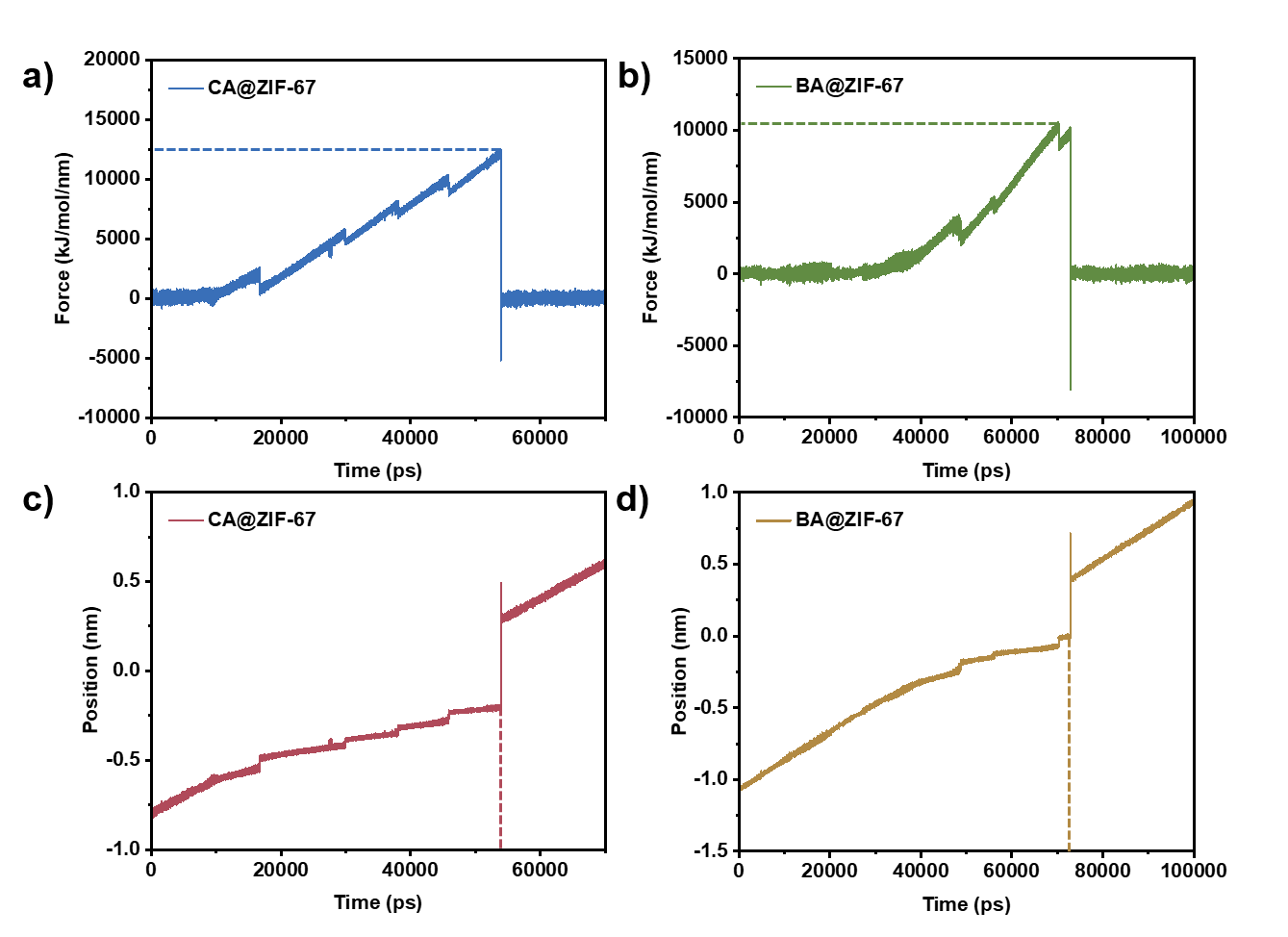


**Figure S7.** a-d) Strength of the force and distance between fragrance molecules and the reference atom group during the non-equilibrium dynamics simulation.


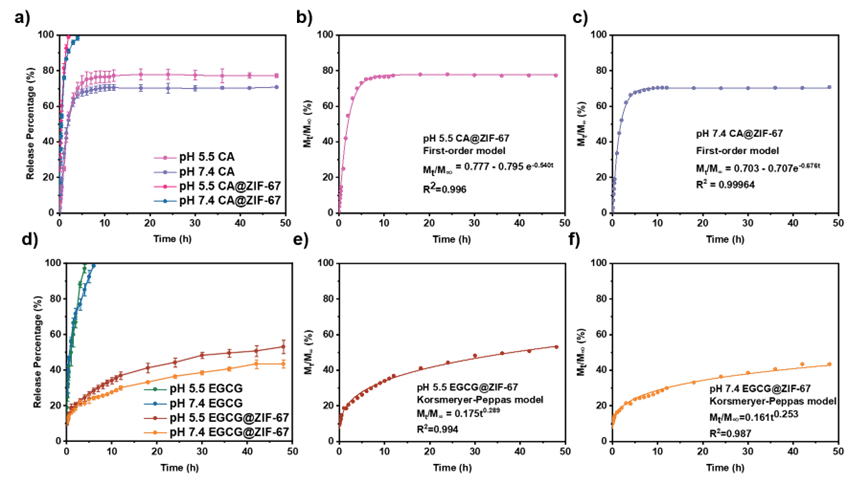


**Figure S8.** a) CA and d) EGCG release profile under pH 5.5 and pH 7.4. b, c) First-order model of CA@ZIF-67 at pH 5.5 and pH 7.4. e, f) Korsmeyer-Peppas release kinetic model of EGCG@ZIF-67 at pH 5.5 and pH 7.4.


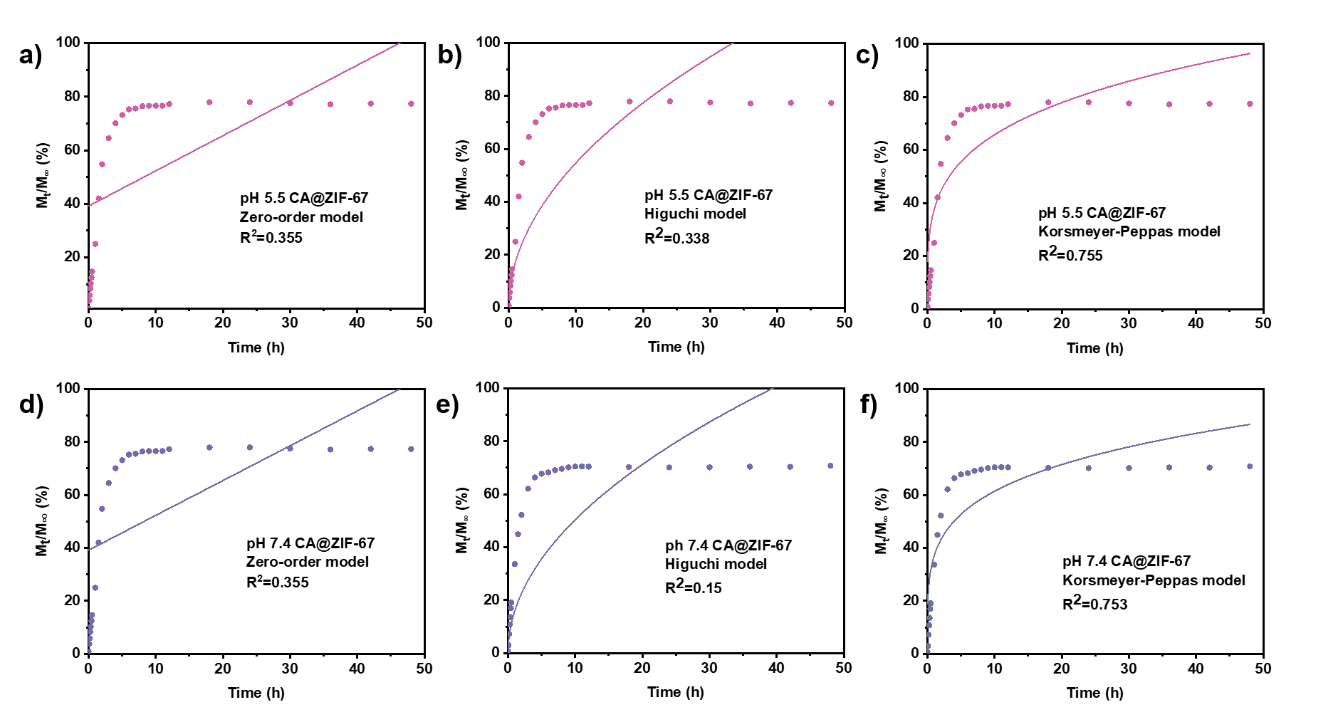


**Figure S9.** a, d) Zero-order model, b, e) Higuchi model and c, f) Korsmeyer-Peppas release kinetic model of CA@ZIF-67 at pH 5.5 and pH 7.4.


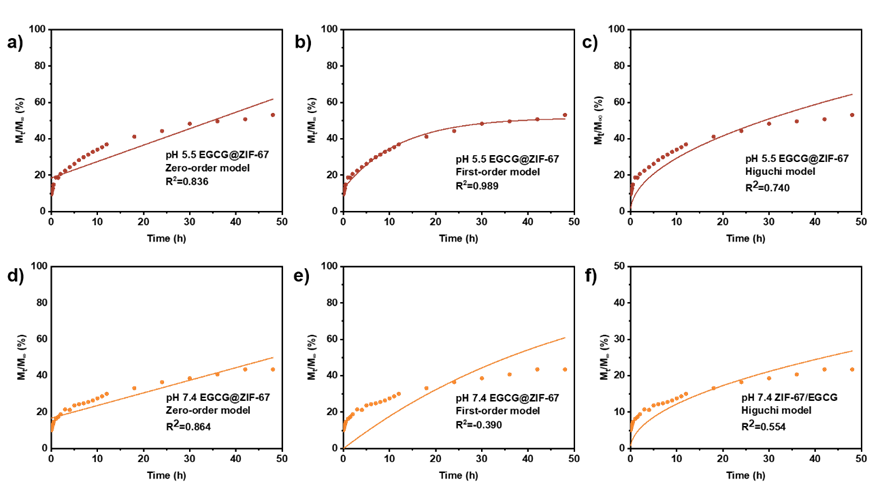


**Figure S10.** a, d) Zero-order model, b, e) First-order model and c, f) Higuchi model of EGCG@ZIF-67 at pH 5.5 and pH 7.4.

**Reference**

(1) Abraham, M. J.; Murtola, T.; Schulz, R.; Pall, S.; Smith, J. C.; Hess, B.; Lindahl, E. GROMACS: High Performance Molecular Simulations through Multi-Level Parallelism from Laptops to Supercomputers. *SoftwareX* **2015**, *1–2*, 19–25. https://doi.org/10.1016/j.softx.2015.06.001.

(2) Jorgensen, W. L.; Tirado-Rives, J. Potential Energy Functions for Atomic-Level Simulations of Water and Organic and Biomolecular Systems. *Proc. Natl. Acad. Sci. U.S.A.* **2005**, *102* (19), 6665–6670. https://doi.org/10.1073/pnas.0408037102.

(3) Krokidas, P.; Castier, M.; Economou, I. G. Computational Study of ZIF-8 and ZIF-67 Performance for Separation of Gas Mixtures. *J. Phys. Chem. C* **2017**, *121* (33), 17999–18011. https://doi.org/10.1021/acs.jpcc.7b05700.

(4) Yang, J.; Zhang, F.; Lu, H.; Hong, X.; Jiang, H.; Wu, Y.; Li, Y. Hollow Zn/Co ZIF Particles Derived from Core–Shell ZIF‐67@ZIF‐8 as Selective Catalyst for the Semi‐Hydrogenation of Acetylene. *Angew. Chem. Int. Ed.* **2015**, *54* (37), 10889–10893. https://doi.org/10.1002/anie.201504242.

(5) Nguyen, T.-B.; Thai, V.-A.; Chen, C.-W.; Huang, C. P.; Doong, R.; Chen, L.; Dong, C.-D. N-Doping Modified Zeolitic Imidazole Framework-67 (ZIF-67) for Enhanced Peroxymonosulfate Activation to Remove Ciprofloxacin from Aqueous Solution. *Sep. Purif. Technol.* **2022**, *288*, 120719. https://doi.org/10.1016/j.seppur.2022.120719.

(6) Qian, J.; Sun, F.; Qin, L. Hydrothermal Synthesis of Zeolitic Imidazolate Framework-67 (ZIF-67) Nanocrystals. *Mater. Lett.* **2012**, *82*, 220–223. https://doi.org/10.1016/j.matlet.2012.05.077.
